# Semantic Representations Are Updated Across the Lifespan Reflecting Diachronic Language Change

**DOI:** 10.1162/OPMI.a.315

**Published:** 2025-12-18

**Authors:** Ellis Cain, Rachel Ryskin

**Affiliations:** Department of Cognitive and Information Sciences, University of California, Merced, Merced, CA, USA

**Keywords:** lexical semantics, language change, learning, aging

## Abstract

Humans learn the meanings of words from the contexts in which they are used. Patterns of language use change over time, suggesting that the contexts in which some words are experienced change across an individual’s lifespan. Here, we investigated whether language users’ semantic space changes in lockstep with changes in the language or whether it retains traces of historical language use/meanings. In two studies, we used distributional semantic word embeddings trained on corpora from different decades (HistWords) to capture meaning change at the level of the (English) language. We first compared these diachronic semantic spaces to the semantic spaces of individuals in different age cohorts (ranging from people in their 20s to people over 70) using an open dataset of associations norms (Small World of Words). Then, using HistWords, we sampled English words that have changed in meaning and words that have maintained the same meaning/usage patterns between the 1950s and the 1990s and collected relatedness judgments for those words with their nearest neighbors from each decade (1950s and 1990s) from both younger (18–33 years) and older (63–92 years) adults. Across the two studies, the semantic spaces of both older and younger adults were most strongly correlated with the semantic spaces derived from more recent corpora. We found little evidence of historical semantic spaces being differentially predictive of the semantic spaces of older adults relative to those of young adults. Our findings suggest that individuals continuously and rapidly update their lexico-semantic representations regardless of age, such that word meanings learned earlier in life are largely replaced with new meanings derived from later language experience.

## INTRODUCTION

Language can be viewed as a complex adaptive system (Five Graces Group et al., 2009), in which global patterns and regularities arise from interactions between individual language users. The behavior of speakers in a speech (or writing/signing) community—how they choose to express their intended messages—is based on social norms, cognitive pressures, and memory for past interactions. From these individual behaviors emerge the collective language usage patterns, which are recorded in corpora. Future individuals learn from past collective usage patterns, as well as from interactions with others in their community. A consequence of this complex adaptive system view is that language usage patterns may change over historical time, across (and even within) generations of language users (Bybee, 2015; Bynon, 1977). For instance, word meanings change over time: broadcast used to be a farming term (i.e., *broadcast the seeds*), icon a religious term (i.e., *a religious icon*), and google a math term, however, now their dominant meanings have changed to more media and computer-centric senses. Individual language users are able to change their lexico-semantic representations and learn these new meanings as they gain prominence. Yet, how meaning change occurs across the lifespan of an individual and how it is related to change in the language at the collective level is an open question.

### Changes in Meaning at the Level of the Language

According to usage based theories of language, word meanings are inextricably linked to the context of their use (e.g., Firth, 1957; Harris, 1954; Wittgenstein, 1953). Words which appear in similar contexts will tend to have similar meanings (e.g., ‘dog’ and ‘cat’). This ‘distributional semantics’ view motivates the use of algorithms like Word2Vec (Mikolov et al., 2013) which use word-context co-occurrence statistics to embed words into a semantic space. These embeddings are known to successfully capture aspects of meaning when compared to human judgments (e.g., Ettinger & Linzen, 2016; Grand et al., 2022; Hill et al., 2015; Lewis et al., 2019).

Diachronic changes in lexical and morphological usage have been documented through corpus analyses (e.g., Davies, 2012; Hilpert & Gries, 2009; Michel et al., 2011), suggesting that the semantic space of the language as a whole may be continually changing. While new words may occasionally be created to capture a new meaning, pre-existing words are often reused and extended to include new meaning senses, leading to polysemy (Ramiro et al., 2018; Srinivasan & Rabagliati, 2015). In other words, polysemy serves as a way for a (limited) lexicon to keep up-to-date with changes in the world or new associations created by language users.

Hamilton et al. (2018) developed a methodology for quantifying semantic change through the evaluation of word embeddings against known historical changes. They trained Word2Vec on decade-level subsets of the Google Ngrams corpus (Michel et al., 2011) and aligned these decade-level embeddings using an orthogonal Procrustes transformation. Their findings suggested that less frequently used words are more likely to undergo meaning change, and secondly, more polysemous words are more likely to undergo meaning change. Polysemy may serve as a driver of semantic change, such that increased flexibility in usage and previous meaning extensions may lead to more extensions in the future. In a parallel analysis of the same corpus, Xu and Kemp (2015) found that related words undergo parallel changes in meaning, with cognitive mechanisms such as analogy preserving the patterns of relationship between words. These semantic changes at the collective level are driven by aggregate changes in individual usage patterns. Yet, it is unknown whether these reflect parallel changes within individuals or changes in population composition.

### Changes in Meaning Across the Lifespan

A common way to model our representational structure of linguistic information is through lexical association networks (e.g., De Deyne et al., 2019; Kumar et al., 2022; Siew et al., 2019), where words are represented as the nodes of a network, and associations between these are represented through edges. The structure of these lexical networks have been found to differ by age. Using word association data, Dubossarsky et al. (2017) found that networks are small and sparse during language acquisition (10–18 years old), they are dense and well-connected in mid-life, and then they become sparse again (though larger overall), with a larger proportion of isolated, peripheral nodes, in late-life. Similarly, Cosgrove et al. (2021) found that the semantic networks of younger adults were more interconnected and resilient to disruption compared to those of older adults, which were sparse and segregated.

One explanation for these differences in lexical-semantic networks across the lifespan is that they are the result of age-related cognitive decline which leads to retrieval difficulties in the word association task (e.g., Hills et al., 2013). Alternatively, the changing structure of lexical semantic networks may be the result of lifelong language learning (Brysbaert, Stevens, et al., 2014; Hartshorne & Germine, 2015; Kuperman et al., 2012). It is important to note that these explanations are not mutually exclusive. Adults continue to engage in statistical learning and acquire novel word-meaning mappings based on regularities in the co-occurrences of novel words and contexts (Fitneva & Christiansen, 2011; Smith et al., 2011; Yu & Smith, 2007). And adults’ vocabularies continue to grow across the lifespan, even as other cognitive functions (e.g., executive function, working memory) begin to decline (e.g. Hartshorne & Germine, 2015). Given the skewed distribution of term frequency in natural languages (Piantadosi, 2014; Zipf, 1935), the network sparsity in older adults may be due to the fact that many individuals may only encounter certain rare words well into adulthood. Similarly, age-related declines in performance in paired associate learning and memory search tasks, previously attributed to cognitive decline, can be explained by the effects of longer learning periods and increased vocabularies in older adults (i.e., Baayen et al., 2017; Ramscar et al., 2014).

Further, Castro et al. (2021) analyzed category exemplar responses (e.g., category: ‘a bird’, exemplar: ‘robin’) across different age groups and time periods. They found that the response patterns to many categories were generally stable (i.e., exemplar frequency was highly correlated across time periods/ages), but the ordering of the responses was not always consistent between generations. Categories that underwent change were likely influenced by historical or social factors (e.g., diseases, toys, weapons, fuels), suggesting that lexical meaning changes at the level of the language may lead to differences in lexical meaning between age groups.

### Language Change and Age-Related Meaning Change

Differences in lexico-semantic representations across age groups could be largely idiosyncratic. Older adults typically know more words than young adults but which additional words they know may differ across individuals, resulting in greater variability in some word meanings for older adults relative to young adults (i.e., Brysbaert et al., 2016). In contrast, it may be that the differences between age cohorts reflect shared prior experiences. Older adults’ language experience differs from that of younger adults not only in the quantity of words but also in the nature of the contexts in which (some) words have historically appeared. For instance, over the period of time during which a 70-year-old has been experiencing and continually tracking language use patterns (in 2025, a 70-year-old would have started experiencing language in the 1950s), the collective usage patterns and meanings of some words have shifted (Hamilton et al., 2018).

In order to be able to coordinate with other members of their community (Chater & Christiansen, 2010; Five Graces Group et al., 2009), individual language users must update their lexico-semantic representations to match the usage patterns of the other members (e.g., learn a new meaning for “tweet”). If the differences in lexico-semantic networks across age groups are driven primarily by changes in language use patterns, there may be systematic differences in some word meanings for older adults relative to younger adults. Specifically, those differences would reflect the historical meanings of those words.

To our knowledge, only one previous study has looked directly at the relationship between diachronic language change and differences in word meanings across age cohorts. Li and Siew (2022) found that words whose meaning changed more over time elicited slower response times in a semantic decision task, but crucially, this effect was stronger among middle-aged adults (45–55 years old) than young adults (18–25 years old). Their results are suggestive of systematic differences in the lexico-semantic representations of older and younger adults that are related to their differential experience of a changing language. However, the meanings of words were probed only indirectly (via a judgment of concreteness), and because the semantic decision task is speeded, it is unknown whether these age-related effects of meaning change impact all aspects of semantic representation and processing or only real-time aspects (e.g., lexical retrieval). Thus, whether there are systematic age-related differences in some word meanings reflecting their historical change remains an open question.

### Present Research

The goals of the present work are to probe differences in lexico-semantic representations across different age groups and test how well these are explained by diachronic language change at the collective level. In two studies, we use representational similarity analysis (RSA; Kriegeskorte et al., 2008) to compare word similarities derived from historical word embeddings (HistWords; Hamilton et al., 2018) across decades to word similarity values obtained from behavioral data from different age cohorts. In the first study, we used openly-available word association data (Small World of Words; De Deyne et al., 2019) to derive word similarities for different age cohorts (from 20 to 70 years of age). In the second study, we collected semantic relatedness judgments from participants in two age groups: younger adults (18 to 33 years of age) and older adults (63 to 92 years of age).

We view the three types of data used for RSA—word embeddings, word associations, and relatedness judgments—as capturing semantic information in distinct but deeply interconnected ways. In everyday language use, speakers and writers draw on their stored representations of meaning, and distributional semantics models are trained on large quantities of aggregated usage data. To the extent that these training data are reasonably representative, the embedding space created by a distributional semantics model will capture the aggregate semantic information in the minds of language users (see Lenci, 2018). Word associations are likely the product of a memory search (i.e., Abbott et al., 2012; Hills et al., 2012, 2015), where individuals generate responses that are associated with a cue by searching through their stored semantic representations. On an individual trial level, the associations are influenced by context, memory search, and expertise (as in response chaining; De Deyne et al., 2019), but once aggregated across a large enough group of trials and people, these patterns of associations can roughly capture the semantic space of a group of language users (Reilly et al., 2025). Relatedness judgments likely require individuals to retrieve and compute the overlap between two words’ semantic representations in the same semantic space (Johns & Jones, 2022), which then must be quantified on a reference scale. Similar to word associations, particular relatedness judgments might be influenced by context and other factors, but they can also be aggregated across a large enough group to represent the relatedness of words in the semantic space of a group of language users. Since words that are related tend to be produced in similar contexts (i.e., Firth, 1957), the relatedness of two words is often tied to linguistic co-occurrence (which is what word embeddings are also based on; Hill et al., 2015; McRae et al., 2012). Therefore, the RSA conducted in the present study is comparing distinct representations of similar semantic information, which will allow us to examine the relationship between the semantic representations of different age groups and historical corpus-based representations.

**Figure 1.**
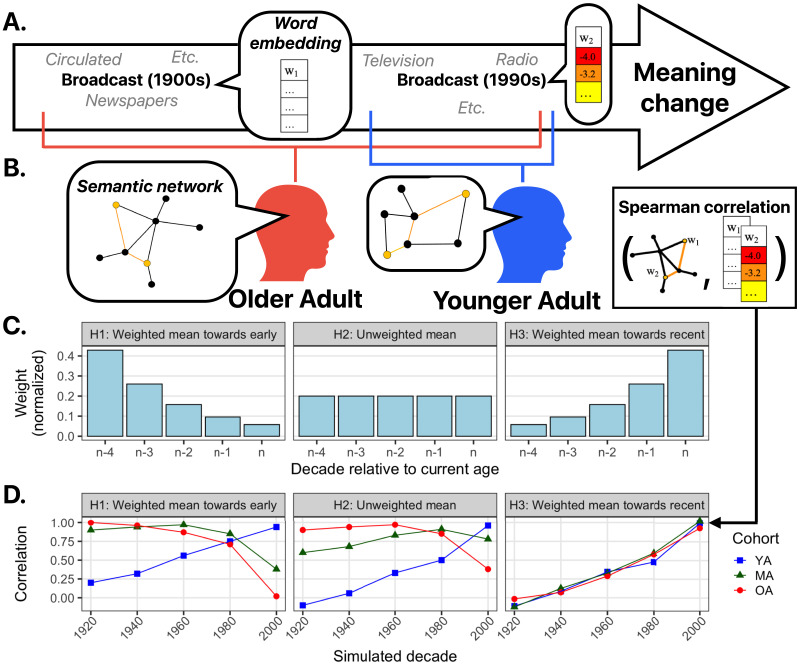
(A) Meaning change as captured by changing nearest neighbors and co-occurring terms. (B) Due to differences in language learning input, different age groups have “experienced” different sets of meaning over time and might have different representations. (C) Different hypotheses for how the language experience might be integrated from across an individual’s lifespan, varying in how different time periods are weighted. (D) Simulated pairwise correlation (RSA) between each age cohort’s similarity values and those from the simulated corpora. (YA = Young adults, MA = Middle-aged adults, OA = Older adults.)

The form of the relationship between similarities from different decades of corpora and similarities from different age groups will depend on how adults update their word meaning representations in response to experiencing new co-occurrence statistics. Figure 1 provides a schematic depiction of potential hypotheses about how meaning change at the level of the language could manifest in different patterns of correlations across age groups. Word meanings change over time, as shown by the turnover in nearest neighbors at different timepoints, and the time-period-specific meanings can be captured using historical word embeddings (Figure 1A). Different age groups will have experienced and learned different meanings of a word that changed (Figure 1B). Figure 1C illustrates three hypotheses about how meanings experienced at different points over the lifespan contribute to an individual’s current meaning representation: all experienced meanings are combined (averaged) and 1) those experienced earlier are weighted more heavily than recent experiences (H1: *weighted mean towards early*)^1^, 2) all experienced meanings are weighted equally (H2: *unweighted mean*), and 3) recently experienced meanings are weighted more heavily (H3: *weighted mean towards recent*)^2^.

Figure 1D simulates the correlations between the meaning representations of individuals from different age groups (e.g., as captured by similarity judgments for words and their neighbors) and the meanings captured in the collective usage patterns of different decades (e.g., semantic similarities of those same words and neighbors from embeddings trained on decade-specific corpora) according to the three hypotheses. According to H1, *weighted mean towards early*, similarity derived from behavioral data from older adults would be most strongly correlated with corpus similarities from earlier decades. According to H2, *unweighted mean*, the correlations for older adults would be strongest for decades in the middle of their lifespan. According to H3, *weighted mean towards recent*, the correlations for older adults would be strongest for the most recent decades. According to all three hypotheses, for young adults the correlations would be highest for recent decades.

## STUDY 1

We aimed to quantify the extent to which diachronic changes in meaning/usage patterns explain age-related differences in lexico-semantic association networks. In order to relate diachronic corpus-based word embeddings and word association data, we used representational similarity analysis (RSA), which uses second-order isomorphic representational similarity matrices (RSMs) to abstract away from the original format of the data^3^. In particular, word embeddings (*n*-dimensional vectors) cannot be directly compared to word association data (cue-response pairs), therefore, we first compute pairwise similarities between words from each data source and represent them in RSMs, which can be directly correlated with one another (for a similar approach, see Ettinger & Linzen, 2016). The embedding-based RSMs are generated separately for each decade and are intended to capture the lexico-semantic information encoded in that decade’s language usage patterns (Harris, 1954; Lenci, 2018). RSMs were generated separately from the word association data from three age cohorts (younger, middle-age, and older adults) and are intended to capture the lexico-semantic representations of each age cohort; they are matrix equivalents of their semantic networks.

**Figure 2.**
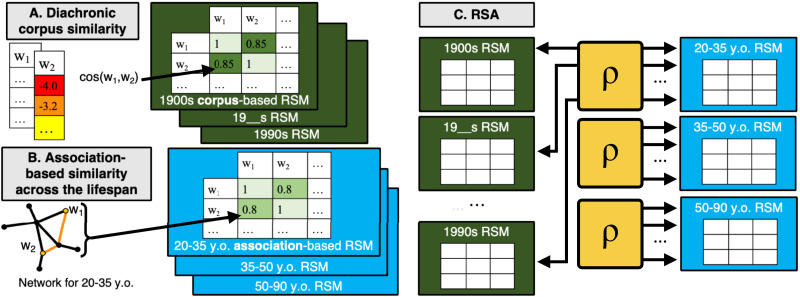
Schematic of data and representational similarity analyses (RSA) in Study 1. (A) For each decade in the diachronic word embeddings, representational similarity matrices (RSM) are generated where each row/column represents a word, and each cell is the cosine similarity between two word embeddings. (B) Using the lexical association networks from Small World of Words, RSMs are generated where each cell is the similarity derived from a Katz random walk function over the network. This is repeated for each age cohort. (C) RSA, implemented as Spearman correlation, is used to compare each RSM from the association data with the RSMs from the diachronic word embeddings.

Figure 2 provides an overview of the analyses: first, we compared corpus-based RSMs across decades to quantify the extent of meaning change over time (Figure 2A); second, we compared the association-based RSMs across age groups to test the similarity of representations across different age cohorts (Figure 2B); third, we conducted RSA between the two sets of RSMs (Figure 2C).

### Methods

#### Diachronic Word Embeddings.

We used the English diachronic word embeddings from HistWords (Hamilton et al., 2018) to construct RSMs for each decade from the 1900s to the 1990s, where each cell represents the cosine similarity between two word embeddings (Figure 2A). The embeddings in HistWords were generated using a Word2Vec model (Skip-gram negative sampling) trained on decade-level subsets of text corpora. We used the English Google N-gram All embeddings as the default because they performed best at predicting known historical changes (Hamilton et al., 2018), but we also replicated the analyses using the Google N-gram Fiction and COHA (Corpus of Historical American English; Davies, 2012) Lemma^4^ embeddings. The embeddings from different decades were aligned using an orthogonal Procrustes method, allowing for direct comparison within and between decades.

#### Lexical Association Networks.

We used English word association data from the Small World of Words (De Deyne et al., 2019) dataset to construct separate RSMs for each age cohort (Figure 2B). Between 2011 and 2018, De Deyne and colleagues collected up to 3 responses for 12,292 cues (such as “couple”, “plug”, “condense”). They had 88,722 participants in total (mean age = 36 years old, SD = 16 years, female = 38%). We split the data into three age cohorts (Younger adults: 20–35 years old, *n* = 14,346; Middle-aged adults: 35–50 years old, *n* = 11,361; Older adults: 50–90 years old, *n* = 6,035).

While the word association data in their original form (cue-response pairs) do not directly map onto similarities between words, De Deyne et al. (2019) developed techniques to estimate semantic similarity using the association data, which are used and described briefly here. First, semantic networks are generated from the group-aggregate word association data, by connecting words/nodes *i* and *j* if any person produced *j* in response to *i* as a cue. This semantic network is then converted to an adjacency matrix (where the rows and columns refer to the terms, and the values to the connection strength), which is then weighted using a positive point-wise mutual information (PPMI) transformation and row-normalized. Then, since the adjusted adjacency matrix corresponds to a random walk transition matrix, decaying random walks are used to add indirect links to a graph. This graph is then PPMI transformed and the values are normalized to conditional probabilities. Finally, cosine similarity is used on the rows from this matrix to calculate the similarity between words. The intuition behind this method is that the random walks capture the indirect relationships between words in addition to the direct links. More details can be found in De Deyne et al. (2016, 2019).

To ensure that there were enough responses per cue from the Small World of Words data, we additionally filtered each RSM to cues that had at least 15 responses per age cohort (*M*_20−35_ = 55.1 responses per cue; *M*_35−50_ = 43.3; *M*_50−90_ = 32.3). Then, for both the HistWords-based and Small World of Words-based RSMs, we restricted the terms to those shared between both datasets, resulting in 6,886 terms^5^.

The word embeddings used for Study 1 are from Hamilton et al. (2018), and the association data are from De Deyne et al. (2019). Scripts for all analyses can be found at https://osf.io/q7j9n/overview?view_only=97f96afc9d614575af607fbc59836afe.

### Results

#### Representational Similarity Analysis.

**Figure 3.**
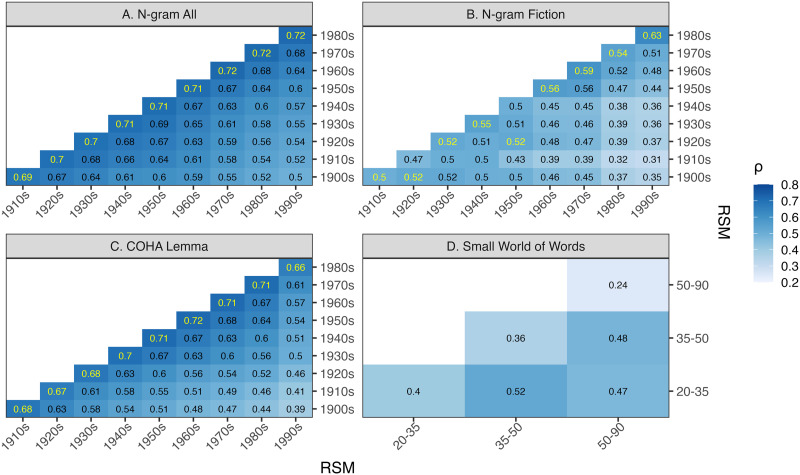
(A–C) Heat-map of RSA between the corpus-based RSMs, for the different versions of HistWords. Each cell represents the Spearman correlation coefficient between two decades. The diagonal was excluded since it would be a perfect correlation. Yellow text is used to highlight the highest correlation in each column. D) Heat-map of RSA between the association-based RSMs. Each cell represents the Spearman correlation coefficient between RSMs of two age groups. The diagonal values represent split-half correlations for the association-based RSA.

**Figure 4.**
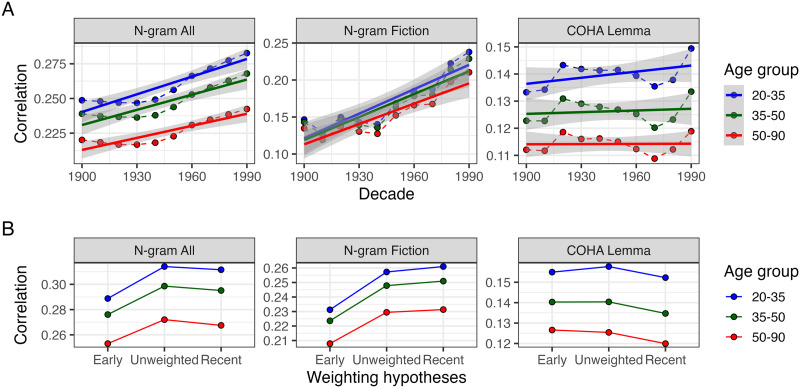
(A) Spearman correlations (RSA) between the association-based and corpus-based RSMs over time, for each corpus-version of HistWords. Each line connects the correlation values for a specific age cohort. (B) Spearman correlation between the association-based and hypothesis RSMs, for each corpus-version of HistWords. Note that the scales differ for each facet of the graph.

We first conducted RSA using Spearman rank correlation to compare the semantic spaces from HistWords across decades (Figure 3A–C), from Small World of Words across age cohorts (Figure 3D), and between the two sources for all decade-age pairs (Figure 2C; Figure 4). In these RSA plots, each cell represents the Spearman correlation between the off-diagonal upper-triangle similarity values from the two RSMs (Ritchie et al., 2017).

##### Diachronic Word Meaning Change in Corpora.

For the corpus-based semantic organization from Google N-gram All (Figure 3A), the RSMs were most similar for adjacent decades (0.69 ≤ *ρ* ≤ 0.72; top value from each column), and as the temporal distance between decades increased, the similarity gradually decreased, with the 1900s and 1990s being the least similar (*ρ* = 0.5). A permutation test indicates that the temporal distance between decades is correlated with the *ρ* value between the RSMs of the same decades (*ρ*_ngram all_ = 0.63, 95% CI = [0.52, 0.72]). For HistWords embeddings trained on other corpora (N-gram Fiction and COHA Lemma), we find similar patterns of temporally close decades having higher correlation (*ρ*_ngram fiction_ = 0.46, 95% CI = [0.33, 0.59]; *ρ*_coha lemma_ = 0.59, 95% CI = [0.41, 0.71]). See Appendix A for further details.

For the N-gram Fiction embeddings (Figure 3B), the closest decades have the highest correlation except for the 1920s and 1950s, which are most correlated with the 1900s (two decades earlier) and 1920s (three decades earlier), respectively. RSMs from COHA Lemma embeddings (Figure 3C) were also more correlated when they were more temporally close, as with N-gram All, but the range of correlations was greater: the most similar RSMs were always for the adjacent decades (0.66 ≤ *ρ* ≤ 0.72), and the temporally distant decades were less similar, with the 1900s and 1990s also being the least similar (*ρ* = 0.39).

##### Change in Lexical-Semantic Representations Across Age Cohorts.

The results of all pairwise correlations between association-based RSMs from different age groups can be seen in Figure 3D. To measure the internal reliability and consistency of the responses for each age cohort subsets, we calculated the average correlation between two RSMs generated from five random splits (in half) of an age cohort’s association data. The internal reliability measures are along the diagonal of the heat-map. The association-based RSMs were less correlated than the HistWords-based RSMs overall (*ρ* ≈ 0.5) and had relatively low internal reliability (*ρ* ≈ 0.2–0.4). These values are likely attenuated because they require splitting the data in half. The internal reliability may be related to the quantity of data contributing to each similarity value. The 20–35 year old cohort had the highest average number of responses per cue (*M* = 55.1 responses per cue) and the highest internal reliability (*ρ* = 0.4), and the 50–90 year old cohort had the lowest average number of responses per cue (*M* = 32.3 responses per cue) and the lowest internal reliability (*ρ* = 0.24). These modest internal reliabilities place an upper bound on the potential correlations involving these data. No gradient change in similarity related to temporal distance in age cohorts was apparent; the correlation between various age groups was roughly around *ρ* ≈ 0.5, regardless of the age difference between the groups. A permutation test indicates that the correlation between differences in age and the correlations between RSMs (*ρ*_swow_ = 0.49) is within the 95% CI [−0.87, 0.87] for correlations obtained when age cohort labels are randomly shuffled (see Appendix A).

##### Similarity Between Semantic Representations From Diachronic Corpora and Age-Specific Association Data.

Figure 4A shows the results of correlations between RSMs for each decade and RSMs for each age cohort, across the different versions of HistWords. For N-gram All, RSMs from all decades were on average most strongly correlated with the youngest age group’s RSM and least correlated with the oldest age group’s RSM (*ρ*_20−35_ = 0.26, *ρ*_50−90_ = 0.23). The same was true for N-gram Fiction (*ρ*_20−35_ = 0.17, *ρ*_50−90_ = 0.15) and COHA Lemma (*ρ*_20−35_ = 0.14, *ρ*_50−90_ = 0.11), though the difference in correlation values between age groups was much smaller for N-gram fiction. Given that the internal reliability of a measure places an upper bound on any potential correlations with other measures, the lower correlations for the older age group RSMs may be explained by their lower internal reliability (see Figure 3D).

Further, for N-gram All, across all of the age cohorts, the correlations slightly increased as the decade of the corpus-based RSM became more recent. The association-based RSMs from all three age groups were more strongly correlated with the corpus-based RSM from the 1990s (*ρ*_(20−35, 1990*s*)_ = 0.28, *ρ*_(35−50, 1990*s*)_ = 0.27, *ρ*_(50−90, 1990*s*)_ = 0.24) than any other decade (e.g., *ρ*_(20−35, 1900*s*)_ = 0.25, *ρ*_(35−50, 1900*s*)_ = 0.24, *ρ*_(50−90, 1900*s*)_ = 0.22). The same was true for N-gram Fiction (*ρ*_(20−35, 1990*s*)_ = 0.24, *ρ*_(20−35, 1900*s*)_ = 0.15), though with greater magnitude and similar correlation values across the age groups. For COHA Lemma, as with N-gram All, the correlations only slightly increased towards more recent decades (*ρ*_(20−35, 1990*s*)_ = 0.15, *ρ*_(20−35, 1900*s*)_ ≈ 0.13), which was consistent across the age groups.

To test our hypotheses about how different meanings might be weighted over a person’s lifespan, we created three weighted combinations of corpus-based RSMs from different decades. The first hypothesis-RSM was a *weighted mean towards earlier*, in that the similarity values were averaged together, with the earlier decades having a higher weight. The next hypothesis-RSM was an *unweighted mean*, in that each decade was given equal weighting. The last hypothesis-RSM was a *weighted mean towards recent*, with later decades having a higher weight. The weights were calculated using *weight* = 0.5^*x*^, where *x* is a decreasing increment for the *weighted mean towards early* hypothesis or an increasing increment for the *weighted mean towards recent* hypothesis. The weights are normalized before calculating the mean RSMs (see Figure 1C for illustration).

Figure 4B shows the RSA for the hypothesis-RSMs and the association-based RSMs from each age cohort. Overall, the averaging (regardless of weighting) seems to increase the correlation between the corpus-based and association-based representations; the association-based RSMs are more correlated with the hypothesis RSMs than any of the single decade RSMs. The 20–35 y.o. age group also has the highest correlation, followed by the 35–50 y.o. group and the 50–90 y.o. group. For the N-gram All versions of HistWords, the *unweighted mean* and *weighted mean towards recent* hypothesis-RSMs have the highest correlation with association-based RSMSs (*weighted mean towards recent*: *ρ*_20−35_ = 0.311; *unweighted mean*: *ρ*_20−35_ = 0.314), and the *weighted mean towards early* hypothesis-RSM has the lowest correlation (*ρ*_20−35_ = 0.29). For the N-gram Fiction version of HistWords, the *weighted mean towards recent* hypothesis-RSM has the highest correlation (*ρ*_20−35_ = 0.261), followed by the *unweighted mean* (*ρ*_20−35_ = 0.257) and *weighted mean towards early* hypothesis-RSMs (*ρ*_20−35_ = 0.23). In COHA Lemma, across age cohorts, all three hypothesis-RSMs are similarly correlated with the association-based RSM, though the recent-weighted hypothesis-RSM is slightly lower (*weighted mean towards early*: *ρ*_20−35_ = 0.155; *weighted mean towards recent*: *ρ*_20−35_ = 0.158; *unweighted mean*: *ρ*_20−35_ = 0.16).

###### Ablation analysis of the relationship between semantic representations from diachronic corpora and age-specific association data.

To further quantify the relative predictive ability of each decade’s similarity values for each age cohort’s word similarities, we used a linear model to predict each age cohorts’ RSM as a combination of the decade-level RSMs and iteratively ablated each decade to quantify its impact via model comparison.

The equation for the full (non-ablated) model was as follows:

$${\text{sim}}_{\text{swow}}=\beta_{0}+\left(\beta_{1}*{\text{sim}}_{1900}\right)+\ldots +\left(\beta_{10}*{\text{sim}}_{1990}\right)+\varepsilon$$

where *sim*_*swow*_ refers to a similarity value from a given age cohort’s RSM, and *sim*_*year*_ refers to the similarity from a given decade’s RSM, derived from HistWords (from the 1900s through the 1990s).

**Figure 5.**
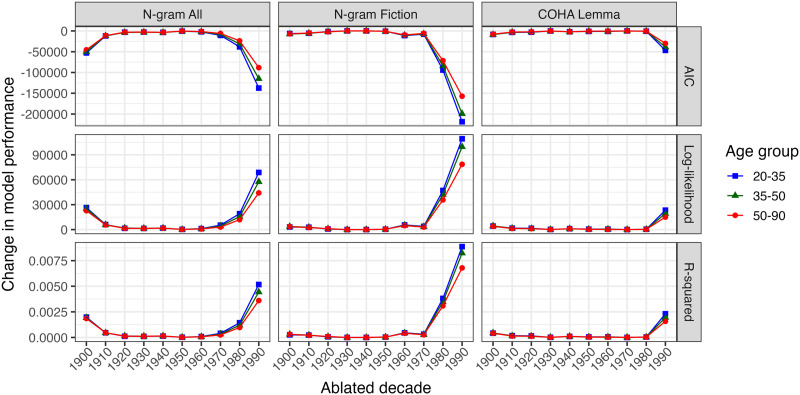
Impact of ablation on model performance for each age cohort, from 1900s–1990s. Each dot represents the difference between full and ablated model performance.

Figure 5 shows the average difference in AIC, Log-likelihood, and *R*^2^ between the full and ablated models across 5 cross-validation folds. The most recent decades have the largest impact on the models’ ability to predict the association-based similarity for all age groups and versions of HistWords. Further, the impact of the most recent decades appears largest for the youngest age cohort and less for the older age cohort.

With the exception of ablating the 1930s or 1940s for the 35–50 year old group, F-tests comparing the model fit of the full and ablated models showed that the full model always predicts the association-based similarity values significantly better than the ablated models (*p*s < 0.001). Appendix B includes the details for the ablation, and the tables with the AIC, log-likelihood, *R*^2^ values, and F-test statistics can be found in the Supplementary Materials.

### Discussion

Corpus-based semantic similarity spaces changed gradually over time. Importantly, non-trivial changes occurr within the lifespan of an older adult living in the 2000s: the correlation between similarities from the 1990s and 1980s was *ρ* = 0.72, whereas the correlation between the 1990s and 1940s was *ρ* = 0.57. Nonetheless, the minimum correlation was *ρ* ≈ 0.5, indicating that while there may be broad changes in linguistic meaning over time, there is substantial stability as well.

In contrast, the association-based semantic similarity spaces appeared somewhat consistent across age cohorts. This may be in part because the cue words selected for Small World of Words were intentionally chosen to be high frequency words with an early average age of acquisition − properties that make a word less likely to change over time (Hamilton et al., 2018). Similarity to the corpus-based meanings decreased with age regardless of the corpus decade, with the 20–35-year-olds having the highest correlation, and the 50–90 years old having the lowest. This reduced correlation with representations derived from corpora is consistent with more idiosyncratic knowledge of the language in older adults. However, caution is warranted in interpreting this result because the internal reliability of the RSMs from older adult data was also lower than that of RSMs from younger adult data, likely due to a smaller average number of responses per cue in the former.

Across age cohorts, association-based semantic representations were most correlated with, and best predicted by, the 1990s corpus-based semantic representations. For all age groups, lexico-semantic representations appear to be best captured by usage patterns from the most recent decades. The results were largely invariant to the choice of embeddings, though the magnitude of the temporal trend was smaller when using the N-gram All and COHA Lemma embeddings (The latter of which had the lowest performance at identifying known changes in meaning; Hamilton et al., 2018). Further, the correlation patterns of the hypothesis-RSMs differed across embedding versions, with the *unweighted mean* and *weighted mean towards recent* having the highest correlation for N-gram All and N-gram Fiction respectively, and the *weighted mean towards early* and *unweighted mean* having the highest correlation in COHA Lemma, depending on the age group.

In sum, the results are most consistent with language users continuing to update their knowledge of word meanings across the lifespan as they experience a changing language. Moreover, recently experienced meanings appear to play a larger role in an individual’s semantic representations than meanings experienced earlier in their life.

## STUDY 2

The dataset of word associations used in Study 1 yielded valuable insights but was limited in multiple ways: the behavioral responses were based on associations as opposed to relatedness (which is what the similarities of HistWords embeddings are thought to represent), the sample sizes across age bins were uneven, and the internal reliabilities of the association-based RSMs were modest. Moreover, the cue words for the Small World of Words dataset were selected to maximize the number of associates produced by participants of all ages. These cues may not be optimal for studying the interaction between language change and age-related differences in lexico-semantic representations.

Therefore, in Study 2, we collected explicit word relatedness judgments (following Gerz et al., 2016; Hill et al., 2015), from both young (18–33 years old) and older adults (63–92 years old), comparing words that changed in their usage/meaning over the approximate lifetime of the older adults (1950 to 2000) to those that didn’t.

### Methods

#### Participants.

1384 English-speaking participants were recruited through the online crowd-sourcing platforms Amazon Mechanical Turk and Prolific. All participants correctly answered 5 catch trials and completed the task in a reasonable time-frame (they took longer than 4 minutes). The catch trials consisted of selecting a word pair that contained a non-word (i.e., “shoe” and “blicket”) from a set of four pairs. Participants were split into two groups based on their age: younger adults (YA ≤ 33 years old, born after ∼1990), older adults (OA ≥ 63 years old; born before ∼1960). We collected data until there were at least 5 ratings for each pair from each group. As a result, the YA group included 730 participants (*M* = 27.7 years old, *SD* = 4.10, 46% Female), and the OA group included 654 participants (*M* = 68.0 years old, *SD* = 4.45, 53.2% Female). Participants took 13.7 minutes on average to complete the task.

#### Materials.

The stimuli for this experiment were pairs of words selected using the HistWords diachronic word embeddings. The target words were drawn from the HistWords vocabulary and filtered by frequency based on SUBTLEX (Brysbaert & New, 2009), such that they included a wide range of usage frequencies (mean log-frequency = 3.28, range = 1.04–4.42).

Since the HistWords embeddings were aligned using an orthogonal Procrustes rotation, we can directly compare the word embeddings across decades to quantify the extent of semantic change. The most recent decade available in the HistWords embeddings is 1990–2000s. The earliest decade in which our participants would have experienced language was the 1950s. By choosing nearest neighbors from these two decades, we were able to identify word pairs that were drastically different for the changed words (i.e., ‘icon’ and ‘brahma’ in the 1950s vs. ‘icon’ and ‘click’ in the 1990s). We used the decade-level word embeddings to identify 1) 150 target words that had undergone the most change in meaning (i.e., cosine similarity between 1950s and 1990s was < 0.35) such as “icon” and “broadcast” and 2) 150 target words that had not undergone meaning change between the 1950s and 1990s (i.e., cosine similarity between 1950s and 1990s > 0.35) such as “daughter” (300 words total). The cutoff value of 0.35 for cosine similarity was chosen based on mean of the distribution of cosine similarities between 1950s and 1990s vectors for 700 words (350 of the most similar and 350 of the least similar) that were sampled from the terms included in Study 1.

The average cosine similarity in the resulting stimulus set was 0.21 (*SD* = 0.078) for the ‘changed’ target words and 0.71 (*SD* = 0.02) for the ‘unchanged’ target words^6^. The average log-frequency was 2.51 (*SD* = 2.80) for the ‘changed’ target words and 3.58 (*SD* = 3.77) for the ‘unchanged’ target words^7^.

For each of the 300 target words, we selected 10 nearest neighbors from the 1950s and 10 neighbors from the 1990s (20 neighbors per word, 6000 word-neighbor pairs). To serve as an unrelated baseline, 10 unrelated words were randomly selected for each target word from the inverse set of its neighbors (3000 non-neighbor pairs). The total stimulus set consisted of 8999 word pairs (one duplicate pair was excluded). The terms (including both cues and responses) had an average log-frequency of 3.63 (*SD* = 4.70) and an average concreteness rating of 3.23 (*SD* = 1.03), derived from SUBTLEX^8^ (Brysbaert & New, 2009; Brysbaert, Warriner, et al., 2014).

For the final dataset, since the number of responses varied across word-neighbor pairs, we sub-sampled from each age cohort such that there were exactly 5 ratings from each cohort per pair, for a total of 89990 ratings (44995 per cohort). The full set of materials is available in data/ at https://osf.io/q7j9n/overview?view_only=97f96afc9d614575af607fbc59836afe.

#### Procedure and Design.

Participants were presented with 75 word pairs and asked to rate their relatedness on a 7-point Likert scale, where 0 was “unrelated” and 6 was “very closely related”. Pairs were randomly organized and presented to participants in groups of 5 pairs. We asked participants to provide their best guess of relatedness for pairs even if they did not recognize any of the words in the pairs, and they indicated on a separate screen if any words were unknown. Participants were randomly assigned to one of 120 counterbalancing lists. Each list consisted of a random set of 25 target words: 12 (or 13 depending on the counterbalancing list) target words that had changed in meaning/usage, according to the HistWords embeddings, and 13 (or 12) that hadn’t changed. Each participant rated a target word 3 times, paired with: 1) one of its nearest neighbors based on the 1950s embeddings, 2) one of its; nearest neighbors based on the 1990s embeddings, and 3) an unrelated, non-neighbor. The order in which the word pairs were presented was randomly generated for each participant.

To ensure that participants were paying attention, there were five catch trials throughout the experiment, where participants were asked to identify the pair with a non-word from a set of five pairs. Participants who did not answer all five of the catch trials correctly were excluded from the analyses.

All de-identified data and analysis code can be found in data/ and scripts/study2/ at https://osf.io/q7j9n/overview?view_only=97f96afc9d614575af607fbc59836afe.

### Results

There were a total of 89,990 ratings overall (44,995 from each age group). Since the nearest neighbors are dependent on meaning change, some of the neighbors for the unchanged pairs were shared between the 1950s and 1990s. We created a subset with the duplicates removed, which dropped the total number of ratings from 89,990 to 79,920^9^. This subset with duplicates removed was used in the subsequent analyses.

We first checked the quality of the participant ratings using split-half reliability and inter-annotator agreement. Split-half reliability was calculated as the average correlation between random splits of the dataset, per age group. The correlations were averaged over 100 iterations. Both age groups had high consistency within their own group (YA: *ρ* = 0.68; OA: *ρ* = 0.7) and across groups (*ρ* = 0.69).

Inter-annotator agreement was calculated as the average correlation between a given rater and the averages of other raters. We repeated this for both the whole dataset and separately for the changed and unchanged target words. The inter-annotator agreement also suggests a high overall agreement (*ρ*_*YA*_ = 0.74, *ρ*_*OA*_ = 0.77), though there was less agreement for the changed pairs than the unchanged pairs (*ρ*_(*YA*,*changed*)_ = 0.52, *ρ*_(*YA*,*unchanged*)_ = 0.79; *ρ*_(*OA*, *changed*)_ = 0.56, *ρ*_(*OA*, *unchanged*)_ = 0.80).

#### Rating Analysis.

**Figure 6.**
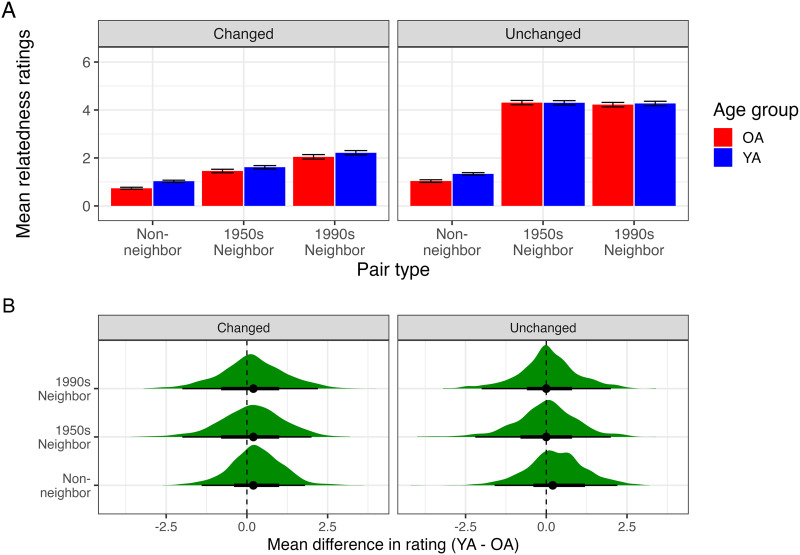
(A) Mean relatedness ratings, from Study 2, by target word type (changed vs. unchanged in meaning from 1950 to 1990) and pair type. Error bars represent 95% confidence intervals over pair means. *N* = 730 YA, 654 OA participants. (B) Distribution of rating difference between the two age groups (YA − OA rating).

Figure 6A shows the average relatedness ratings for the three types of pairs based on whether or not the target word changed in meaning, for both age groups. Figure 6B shows the distribution of differences between the average YA and OA rating across pairs. The differences are on average slightly larger for the changed word pairs than the unchanged word pairs, and the former distribution has a slightly larger spread as well.

We used the brms package in R (Bürkner, 2017) to fit a Bayesian multilevel ordinal model to the relatedness ratings for each target-neighbor pair. We computed estimated marginal means (EMM) and pairwise contrasts using the emmeans package in R (Lenth, 2024). Age group (OA vs. YA), pair type (1990s, 1950s, or non-neighbor), meaning change (Changed vs. Unchanged), and their interactions constituted the fixed predictors^10^. Age group and meaning change were effects coded, and pair type was dummy coded. The YA group, unchanged target words, and non-neighbor pairs were coded as the reference levels for the corresponding variables^11^.

Posterior distributions for model parameters are summarized in Appendix F. Averaging over age groups, the pairs for the changed target words were rated as less related than the pairs for the unchanged target words (Δ_*Non*–*neighbor*_ = −0.68, 95% HPDI = [−0.83, −0.53]; Δ_1950*s*_ = −4.19, 95% HPDI = [−4.37, −4.01]; Δ_1990*s*_ = −3.18, 95% HPDI = [−3.25, −3.01]). For the unchanged target words, the 1950s and 1990s neighbor pairs had similar ratings (*EMM*_1950*s*_ = 4.39, 95% HPDI = [4.19, 4.58]; *EMM*_1990*s*_ = 4.33, 95% HPDI = [4.11, 4.52]) and were both rated as more highly related relative to the non-neighbor pairs (*EMM*_*Non*–*neighbor*_ = −0.39, 95% HPDI = [−0.48, −0.31]). For the changed target words, the non-neighbor pairs also had lower ratings across age groups (*EMM*_*Non*–*neighbor*_ = −1.07, 95% HPDI = [−1.25, −0.91]) relative to the neighbor pairs (*EMM*_1950*s*_ = 0.21, 95% HPDI = [0.03, 0.37]; *EMM*_1990*s*_ = 1.15, 95% HPDI = [0.98, 1.33]).

For both changed and unchanged pairs, older adults rated non-neighbor pairs as less related than young adults did (*β*_*OA*_ = −0.78, 95% CrI = [−0.96, −0.61]). There was an interaction between age group and meaning change, such that, for non-neighbor pairs, the difference between the ratings of YA and OA was smaller for the unchanged than the changed pairs (*β*_*OA*:*Changed*_ = −0.15, 95% CrI = [−0.27, −0.04]). Age group also interacted with pair type, such that, collapsing across changed and unchanged pairs, the difference between OA and YA ratings was reduced for 1950s and 1990s neighbor pairs relative to non-neighbors (*β*_*OA*:1950*s*_ = 0.85, 95% CrI = [0.66, 1.04]; *β*_*OA*:1990*s*_ = 0.77, 95% CrI = [0.59, 0.95]).

There were three-way interactions between age, meaning change, and pair type (*β*_*OA*:*Changed*:1950*s*_ = −0.34, 95% CrI = [−0.53, −0.15]; *β*_*OA*:*Changed*:1990*s*_ = −0.22, 95% CrI = [−0.39, −0.04]): The rating difference between non-neighbors and 1950s/1990s neighbors was larger for older adults than young adults with unchanged words (Older adults: Δ_*Non*–*neighbor*–1950*s*_ = −5.21, 95% HPDI = [−5.44, −5.00]; Δ_*Non*–*neighbor*–1990*s*_ = −5.11, 95% HPDI = [−5.33, −4.89]; Young Adults: Δ_*Non*–*neighbor*–1950*s*_ = −4.36, 95% HPDI = [−4.55, −4.16]; Δ_*Non*–*neighbor*–1990*s*_ = −4.33, 95% HPDI = [−4.52, −4.13]), but less so for changed pairs (Older Adults: Δ_*Non*–*neighbor*–1950*s*_ = −1.53, 95% HPDI = [−1.71, −1.35]; Δ_*Non*–*neighbor*–1990*s*_ = −2.50, 95% HPDI = [−2.68, −2.31]; Young adults: Δ_*Non*–*neighbor*–1950*s*_ = −1.02, 95% HPDI = [−1.18, −0.87]; Δ_*Non*–*neighbor*–1990*s*_ = −1.94, 95% HPDI = [−2.10, −1.78]). However, crucially, for words that changed in meaning, the difference in relatedness ratings between 1950s and 1990s neighbor pairs was similar across the two age groups (OA: Δ_1950*s*–1990*s*_ = −0.96, 95% CrI = [−1.13, −0.79]; YA: Δ_1950*s*–1990*s*_ = −0.92, 95% CrI = [−1.08, −0.77]).

#### Representational Similarity Analysis.

We then conducted RSA using Spearman-rank correlation to compare the semantic spaces of the participants to those derived from HistWords across different decades. For the unchanged words in N-gram All, relatedness ratings with 1950s and 1990s neighbors were positively correlated with cosine similarities from the 1950s (*ρ* = 0.30) and 1990s embeddings (*ρ* = 0.32) respectively. For changed words in N-gram All, relatedness ratings with 1950s neighbors were negatively correlated with cosine similarities from the 1950s (*ρ* = −0.14), and relatedness ratings with 1990s neighbors were not correlated with cosine similarities from the 1990s (*ρ* = 0.03). The correlations did not differ by age group (see Appendix G for visualizations and additional details).

**Figure 7.**
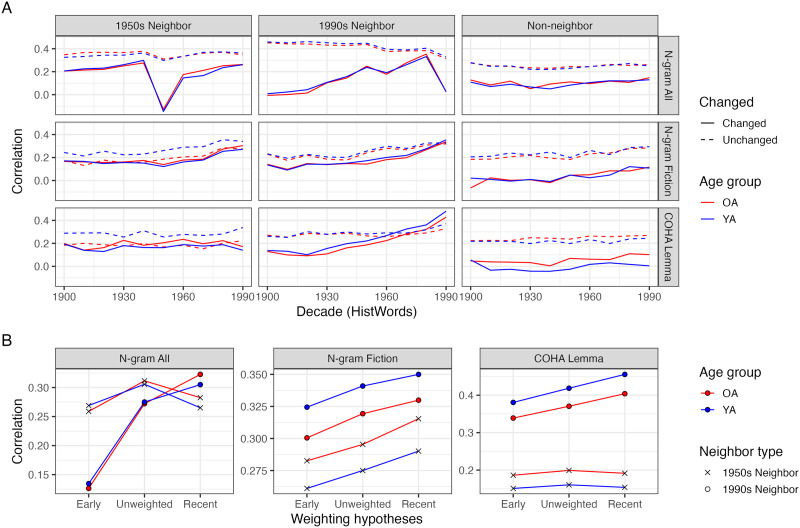
(A) Spearman correlations (RSA) between the relatedness ratings and HistWords similarity over time. Each line connects the correlation values for a specific age group, separately for meaning change and neighbor type. (B) Spearman correlation between the relatedness ratings and hypothesis-RSMs for the changed words, across each corpus-version of HistWords.

Figure 7A summarizes the correlations of the ratings with cosine similarities from all decades of HistWords embeddings starting from the 1900s for both age groups, split by neighbor type, across the different versions of HistWords. In N-gram All, the correlations for the unchanged pairs are stable across decades for both age groups and they are much higher (0.30 ≤ *ρ*_1950s neighbor_ < 0.36; 0.33 ≤ *ρ*_1990s neighbor_ < 0.42; 0.26 < *ρ*_*Non*–*neighbor*_ ≤ 0.32) than for changed pairs (−0.11 < *ρ*_1950s neighbor_ < 0.36; 0.005 < *ρ*_1990s neighbor_ < 0.45; 0.07 < *ρ*_*Non*–*neighbor*_ < 0.20). For the changed 1990s pairs, the correlation generally increases towards more recent decades, with the 1980s decade having the highest correlation to both age groups’ ratings (*ρ* ≈ 0.36), whereas for the changed 1950s pairs, the 1940s decade has the highest correlation to the relatedness ratings (*ρ* ≈ 0.28). In N-gram Fiction, the correlations for the unchanged pairs are also stable across decades for both age groups (0.13 < *ρ*_1950s neighbor_ < 0.36; 0.17 < *ρ*_1990s neighbor_ < 0.33; 0.18 < *ρ*_*Non*–*neighbor*_ < 0.30), and their correlation ranges overlap with the changed 1950s and 1990s pairs but not the non-neighbors (0.12 < *ρ*_1950s neighbor_ < 0.31; 0.09 < *ρ*_1990s neighbor_ < 0.36; −0.01 < *ρ*_*Non*–*neighbor*_ < 0.13). The changed 1990s pairs have the most pronounced temporal trend, with the 1990s embeddings having a higher correlation than the 1900s embeddings (*ρ*_1990s embeds_ ≈ 0.34 > *ρ*_1900s embeds_ ≈ 0.13). In COHA Lemma, the changed 1990s neighbors had a sharp increase in correlation towards the more recent decades (0.09 < *ρ*_1990s neighbor_ < 0.48). The other types on average have stable, moderate correlations (*ρ* ≈ 0.18), with the exception of the changed non-neighbor pairs, which were not correlated (*ρ* ≈ −0.005) Across the different versions, there do not seem to be large differences in correlations between the age groups, with the exception of the unchanged 1950s neighbor pairs in N-gram Fiction and COHA Lemma, where the younger adults’ ratings have slightly higher correlation, and changed non-neighbor pairs in COHA Lemma, where the older adults’ ratings have a slightly higher correlation.

As in Study 1, three hypothesis-RSMs were constructed for each age group based on weighted combinations of HistWords embeddings. Figure 7B shows the RSA for the hypothesis-RSMs and the ratings-based RSMs from each age group, for the changed words and their 1950s and 1990s neighbors. Across all the different versions of HistWords, the *weighted mean towards recent* RSM has the highest correlation with the RSM for the 1990s neighbor pairs. For the 1950s neighbors, the trend differs based on the HistWords training corpus: the different weighting hypotheses appear to be similarly correlated to ratings-based RSMs for COHA Lemma and N-gram All (though the *unweighted mean* hypothesis-RSM has a slightly higher correlation for the 1950s neighbors in N-gram All), while the *weighted mean towards recent* hypothesis-RSM is most correlated with the ratings-based RSMs for the N-gram Fiction version of HistWords.

#### Ablation Analysis.

As in Study 1, we used a model ablation approach to quantify the relative predictive ability of each decade’s similarity values for the two age cohorts’ relatedness ratings. We focused on the 1950s and 1990s neighbor pairs for both changed and unchanged words.

We trained Bayesian multilevel ordinal models to predict participant relatedness ratings as a combination of similarity values from HistWords from 6 decades (1940s–2000s). The full models contained all 6 decades’ similarity values as predictors.^12^ We then constructed reduced models by ablating individual decades to quantify their impact via model comparison with the full model.

We separately trained models for the two types of meaning change (changed and unchanged), since the range of similarity values greatly differed between the two (Changed: *0 < cosine similarity < 0.25*, Unchanged: *0.65 < cosine similarity < 0.75*). Likewise, we separately trained models for the younger and older adults’ ratings for the two different pair types. This resulted in 56 models total (7 ablations × 2 age groups × 2 neighbor types × 2 meaning change types)^13^.

**Figure 8.**
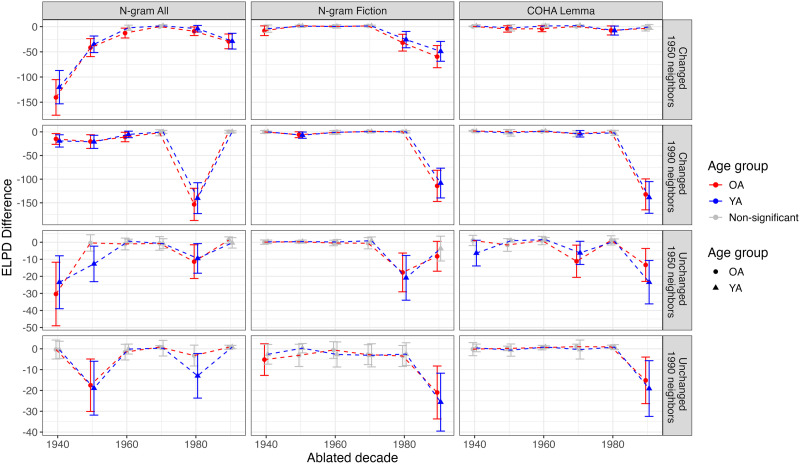
Ablation impact on model performance as measured by the ELPD difference. The four panels show the neighbor types (1950s and 1990s) and meaning change (Changed and Unchanged) for the two age groups. ELPD difference is always relative to the full model. Error bars represent 2 × standard error of the ELPD difference. Ablated models with an ELPD difference of less than four are colored gray. Note that the *y*-axis scale differs across rows.

To quantify the impact of each predictor, we used the loo_compare() function in R to calculate the expected log predictive density (ELPD) difference between each ablated model and the corresponding full model. Figure 8 shows the ELPD difference across the different models^14^. ELPD estimates the predictive performance of a model for new observations using leave-one-out cross-validation. A rule of thumb is that if the ELPD difference between two models is less than four, then the difference between the models is not significant (or the estimation may be poorly calibrated). If the ELPD difference is greater than four, then it should be compared against the standard error (Sivula et al., 2020).

Across the different versions of HistWords and combinations of meaning change and neighbor type, the impact of ablating each decade is similar across the two age groups. The confidence intervals around the ELPD difference overlap between age groups, indicating that there is broad consistency in the relationship between HistWord similarity values and human ratings between age groups.

In N-gram All, for the 1950s changed pairs, ablation of the earliest decades (1940, 1950) has the largest impact on model performance, followed by the most recent decade (1990), with ablation of the middle (1960–1980) decades resulting in small differences. For the 1990s changed pairs, the similarities from the 1980s have the largest impact, followed by those from the 1950s and 1940s. Surprisingly, ablating the 1990s does not seem to have an impact on model performance. Similar to the changed words, for the 1950s pairs of unchanged words, ablation of the earliest decades (1940 and 1950) has the largest impact on ELPD. The ablation impact of the 1950s similarities is numerically larger for young adults than for older adults. Moreover, ablation of the 1980s similarities also has an impact on model performance, consistent across both age groups. For the 1990s unchanged pairs, the 1950s have the largest impact on model performance for both age groups, followed by the 1980s, though the ablation impact is numerically larger for young adults than it is for the older adult group.

For both N-gram Fiction and COHA Lemma, ablation of the most recent decade (1990s) generally has the largest impact. However, for the 1950s changed pairs when using COHA Lemma embeddings, ablating the 1980s has the largest impact, though there appears to be very little impact of any ablation. Similarly, for the 1950s unchanged pairs, when using N-gram Fiction embeddings, ablating the 1980s has the largest impact.

### Discussion

With the exception of the unchanged neighbor pairs, the younger adults rated word pairs as more related than the older adults did, on average. Crucially, for words that changed in meaning between 1950 and 1990, ratings of 1990s neighbors relative to 1950s neighbors did not differ by age. Neighbor pairs from the 1950s were not judged as comparatively more related by older adults than young adults. RSA indicated that ratings of the unchanged pairs were consistently correlated with similarities from all decades of HistWords across the different neighbor types and versions of embeddings. For the changed pairs, ratings of the 1990s neighbors were generally most correlated with similarities from more recent decades of HistWords. This pattern was consistent across the different versions of HistWords but reduced for the 1950s neighbors and non-neighbors.

For words that changed in meaning between 1950 and 1990, the similarity from recent decades contributed most strongly to predicting the relatedness ratings of words with their neighbors from the 1990s, whereas for the neighbors from the 1950s, it was the earliest decade that contributed the most for N-gram All, while the more recent decades contributed the most for N-gram Fiction and COHA Lemma. For words that didn’t change in meaning, both the early and recent decades contributed similarly to predicting current relatedness ratings for both 1950s and 1990s neighbors, highlighting that the meanings of these words have been stable between the 1950s and 1990s. Importantly, this was the case for both older adults and young adults to a similar extent.

**Figure D2.**
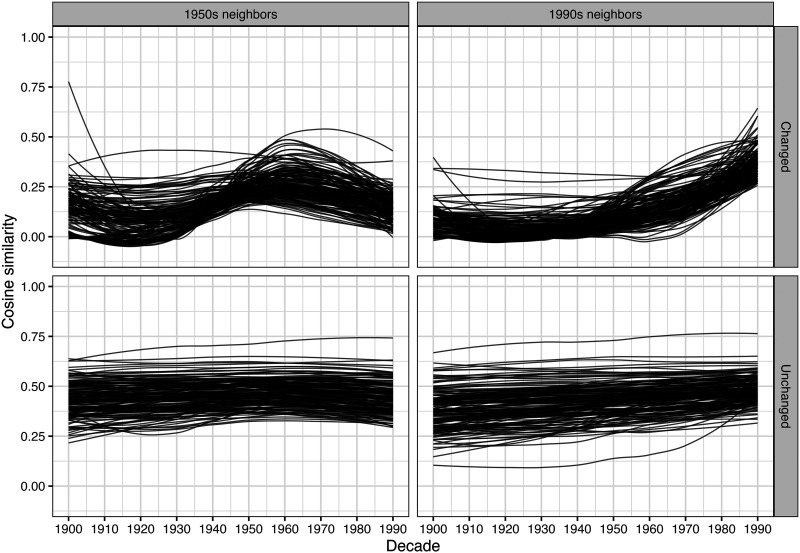
Average cosine similarity between a target word and the nearest neighbors. Vertical facets indicate the neighbor source, whether 1950s or 1990s decades. Each line represents the average cosine similarity between a target word and its set of neighbors.

It is noteworthy that, using N-gram All, the similarities from the 1940s and the 1980s had the largest impact for the 1950s and 1990s neighbors respectively, as opposed to the 1950s and 1990s themselves. This pattern is also seen in the correlation values from Figure 7. This may be related to our stimulus selection process which searched for words which minimized the cosine similarity between the 1990s and 1950s decade embeddings and may have picked out some words with “outlier” meanings in one of those two decades, though the exact reason for this pattern is unclear. Appendix Figure D2 suggests that the 1950s represent an important inflection point for some words in our stimulus set, such that the similarity with their semantic neighborhood (chosen based on the 1950s embeddings) began to rise in the 1940s, peaked around the 1950s and 1960s, and declined in the 1970s. This may reflect some words taking on a temporarily popular sense that then falls back out of favor by the 1980s and 1990s. When young and older adults encounter those neighbor pairs from the 1950s, their judgments of relatedness do not reflect those temporary meanings and even appear to be more similar to some aspects of earlier meanings (from the 1940s and before). Similarly, the semantic neighborhoods from the 1990s for some words may have been outliers (e.g., due to temporarily prominent senses or due to random variability in the composition of the training data) and the current uses of the words may be closer to those captured by the 1980s similarities.

In sum, we observed little difference in the relatedness judgments between age groups and the RSA and ablation analyses indicate that word-neighbor similarity values derived from corpora from different decades contribute similarly to predicting current relatedness ratings of young and older adults. The corpus-based semantic representations from recent decades were more correlated with human ratings in the RSA and had a larger impact in the ablation analysis. Additionally, the *weighted mean towards recent* hypothesis-RSM was more strongly correlated with human ratings than the *unweighted mean* or *weighted mean towards early* hypothesis-RSMs across most corpora and conditions. These findings are largely consistent with the predictions of the *weighted mean towards recent* hypothesis, according to which lexico-semantic representations are continuously updated across the lifespan and evidence from recent linguistic experiences is weighted most heavily when making judgments about word meaning.

## GENERAL DISCUSSION

In the first study, we found that the lexico-semantic representations derived from diachronic corpora changed from one decade to the next, such that the collective language use patterns experienced by different age groups are measurably different. Yet, for all age groups, their lexico-semantic representations (based on word association behavior) were most closely related to the usage patterns of the most recent decade.

Some patterns were suggestive of age-related differences in these diachronic effects: the correlations to corpus-based semantic organization generally decreased with age (i.e., the 50–90 year old group had the lowest correlation across word embedding types). One interpretation is that, as a result of experiencing a larger variety of meanings and usage patterns, the lexico-semantic representations of older adults may become less closely tied to the usage patterns of any particular decade. However, caution is warranted in interpreting these results given the low internal reliability of the association-based similarity measures.

In the second study, we demonstrated that words which changed in meaning between the 1950s and 1990s, according to diachronic embeddings, were judged to be more related in meaning to neighbors from the 1990s than the 1950s. This was not the case for words which didn’t change in meaning: neighbors from the 1950s and 1990s were judged to be equally semantically related, even when we controlled for duplicates and familiarity. Crucially, both younger and older adults judged the words that changed in meaning to be closer to their 1990s neighbors than their 1950s neighbors to the same extent. As in Study 1, the lexico-semantic representations of both age groups appeared to be more closely aligned with the usage patterns of more recent decades, matching the *weighted mean towards recent* hypothesis. The older adults overall provided lower ratings than the young adults. This pattern is potentially analogous to what was observed in Study 1: as a result of experiencing a larger variety of meanings/usage patterns, the lexico-semantic representations of older adults may become less closely tied to the usage patterns of any particular decade. However, the influence of earlier language use patterns—which older adults, but not younger adults, would have experienced in their lifetime—appeared to be similar for both age groups.

Specifically, for the target words that changed in meaning, their present-day relationship to semantic neighbors from the 1950s was best predicted when usage patterns/similarities from earlier decades in N-gram All (1940s and 1950s) were taken into account and their present-day relationship to semantic neighbors from the 1990s was best predicted when usage patterns/similarities from a recent decade in N-gram All (1980s and 1990s) was taken into account. For the target words that didn’t change in meaning, similarities from both early and later decades were important for predicting present-day neighbor relatedness. This suggests that knowledge of past word meanings is maintained across generations, even as usage changes, and may reflect the fact that new senses of a word tend to evolve by branching off of existing meanings rather than by forming entirely *de novo* semantic links (Ramiro et al., 2018; Srinivasan & Rabagliati, 2015).

Across the two studies, differences between age groups were minimal, indicating that, despite much more extended language experience over a time period during which many words changed in meaning, older adults’ lexico-semantic representations primarily reflect present-day usage patterns, as do those of young adults. More specifically, the minimal age-related differences suggest that people rapidly adapt to changes in linguistic meaning and continually track the collective usage patterns, rather than being biased by the prior usage patterns they experienced. The current results are consistent with previous findings that adults continue to update their language representations well into adulthood (Brysbaert, Warriner, et al., 2014; Kuperman et al., 2012; Maciejewski et al., 2020; Rodd et al., 2016; Ryskin et al., 2017) and with a view of semantic representations as being rapidly and continuously updated through (Elman, 2009; Kamath et al., 2025; Rodd, 2020).

### Limitations and Future Directions

An alternative possibility is that lexico-semantic representations do differ between age groups but these differences were simply not detected in our studies for a number of possible reasons. In Study 1, the words in the Small World of Words dataset may have changed minimally in meaning over the lifespan of the participants. Within a typical individual’s lifetime, there is substantial stability in meanings (see Figure 3A; *ρ*_1950–1990_ = 0.6 vs. *ρ*_1980–1990_ = 0.7) and the words selected for the cued-association task may have contained a higher proportion of stable meanings. A more varied set of words may have revealed differences between age groups.

In contrast, the stimuli for Study 2 were selected on the basis of meaning change but this resulted in a large number of target words with lower frequency (*M*_*Freq*_ = 310 in SUBTLEX); more frequent words are less likely to change in meaning (Hamilton et al., 2018). Relatedness ratings for words that changed in meaning with their semantic neighbors (from either decade) were fairly low (*EMM*_*Changed*,1950_ = 0.21, 95% HPDI = [0.033, 0.37]; *EMM*_*Changed*,1990_ = 1.15, 95% HPDI = [0.98, 1.33]). Participants may not have been familiar with all these words, leading to increased rates of guessing. However, excluding data for which participants reported not knowing one of the words did not affect the main results^15^.

Further, the present work assumes that HistWords embeddings adequately capture the statistics of everyday language exposure for most individuals in a given decade. Similarities computed from these embeddings can be expected to explain variance in behavioral measures of meaning similarity to the extent that this assumption is justified. Many aspects of language input which contribute to language users’ meaning representations (e.g., spoken usage, visual context) are absent from the text corpora on which these embeddings are trained, and not all training corpora are equally representative of the average individual’s written language input. For instance, some researchers have pointed out issues with the Google Books corpus, such as the increased inclusion of scientific texts in recent decades, which may bias the lexicon (Pechenick et al., 2015). Despite these limitations, in previous work, the usage statistics present in text corpora have been shown to capture many aspects of meaning representations in individuals (e.g., Ettinger & Linzen, 2016; Grand et al., 2022; Hill et al., 2015). And, in the present work, across the different versions of HistWords trained on different corpora (N-gram All, N-gram Fiction, and COHA Lemma), we generally found similar patterns across decades and cohorts, though effect sizes were reduced when using COHA Lemma. COHA, which is a genre-balanced corpus, might be more representative of ‘typical’ language exposure, but the smaller size might not capture enough usage statistics for a distributional semantics model (i.e., word2vec) to track changes in meaning. The performance of known meaning change detection was lower for COHA than for N-gram All (87.5% vs. 100%; Hamilton et al., 2018). Embeddings trained on larger, more representative corpora may explain more variance in human ratings and provide more accurate estimates of the contributions of meanings from different decades.

Similarly, the context-free nature of the relatedness ratings may have obscured some subtler nuances of meaning due to word-sense extension (e.g., Ramiro et al., 2018) that may differ across language users of different ages. The type of word-pair relatedness ratings used in the present work has been widely used in previous research relating human semantic judgments to distributional semantics models (i.e., Ettinger & Linzen, 2016; Gerz et al., 2016; Hill et al., 2015). However, the isolated word pairs lack context for interpreting the word meanings. For example, ‘unicorn’ may be judged as highly related to ‘horse’ in the context of a discussion of animals, but not in the context of a business discussion (where it denotes a uniquely successful startup company). While the former meaning is known to both older and younger adults, the latter may only be familiar to middle-aged or younger individuals. But, in the absence of context, both younger and older adults may focus on the more established, animal-related meaning. Similarly, the embeddings used in the present work are context-free; there is one embedding per word. The nearest neighbors are, therefore, likely to reflect primarily the more dominant meanings. Future studies could use more diverse neighbor pairings (i.e., to probe non-dominant senses) or contextualized embeddings from language models (e.g., BERT; Devlin et al., 2019; Periti & Tahmasebi, 2024; Qiu & Xu, 2022) and explore how context-embedded rating methods (e.g., Goldstone, 1994, Goldstone et al., 1997) could be used to probe potential age-related differences in representations of polysemous words that have changed in meaning.

Finally, the RSA approach allows us to compare the similarity patterns across a large number of words between data sources that might not have the same format (e.g., word embeddings and association data), but it ignores potentially interesting patterns of individual change for specific words. As mentioned before, taking the language as a whole, meanings within a population of language users change slowly over an individual’s lifespan, but individual words can change abruptly (e.g., ‘tweet’ or ‘broadcast’). It might be that there is a subset of these abruptly-changing words with systematically different meanings for older adults and younger adults. We leave it to future work to identify such a subset and explore whether any age-related differences are the result of different amounts of exposure to a changing language or other differences in language input between younger and older adults (e.g., different genre preferences).

## CONCLUSION

Collective patterns of language use change over time corresponding to diachronic changes in the meanings of words. While younger and older individuals systematically differ in the cumulative usage patterns that they have experienced, the present findings indicate that they rapidly update their lexico-semantic representations to primarily reflect current meanings.

## FUNDING INFORMATION

This research was funded by the National Institutes of Health - National Institute on Aging grant R15 AG073948 to RR.

## AUTHOR CONTRIBUTIONS

E.C.: Conceptualization, Data curation, Formal analysis, Investigation, Methodology, Resources, Software, Validation, Visualization, Writing – original draft, Writing – review & editing. R.R.: Conceptualization, Data curation, Formal analysis, Funding acquisition, Investigation, Methodology, Project administration, Resources, Supervision, Validation, Writing – review & editing.

## DATA AVAILABILITY STATEMENT

Word embeddings used in Studies 1 and 2 are from Hamilton et al. (2018). Word association data used in Study 1 are from De Deyne et al. (2019). Word relatedness judgments used in Study 2 can be found at https://osf.io/q7j9n/overview?view_only=97f96afc9d614575af607fbc59836afe.

## Notes

^1^ This could be understood as meaning change being a *generational* phenomenon: one generation initially learns a word meaning and mainly retains that meaning throughout their lifespan, then the same thing happens with the next generation. Population-level changes in word meaning usage would be tied to changes in population composition.^2^ For simplicity we only consider weights that change monotonically (either increasing or decreasing) with time. Other learning functions are possible as well. For instance, it may be the case that experience from early adulthood is weighted more heavily than that from both the earliest years and the most recent years in older adulthood.^3^ The RSA approach could be understood as converting a geographic map, which contains points of interest (or nodes), from a graphical representation to a distance matrix, where each entry in the matrix represents the distance between two points of interest (or two nodes). If multiple people draw maps of the world, their maps might be different sizes and the outlines of countries may look different, making a pixel-by-pixel comparison difficult. However, the relative distances between nodes should be similar. Representing the maps using distance matrices abstracts from the original format and allows for direct comparison of relative distances, such that one could evaluate the similarity of the maps.^4^ We only used the COHA Lemma version instead of both the Lemma and Word versions as they were trained on the same underlying corpus.^5^ We tried to maximize the number of terms that were included in the analysis of the Small World of Words data, while also excluding cues that had lower numbers of responses, and so we did not select for specific psycholinguistic properties. The average term log-frequency was 3.74 (*SD* = 4.67) and average concreteness rating was 3.33 (*SD* = 1.08), as derived from SUBTLEX (Brysbaert & New, 2009; Brysbaert, Warriner, et al., 2014). The frequency and concreteness data from SUBTLEX covered 95% of the included terms in Study 1.^6^ See Appendix D for details about the distributions of similarity values in both conditions.^7^ This difference in frequency follows previous findings such as Hamilton et al. (2018), which found that lower frequency terms are more likely to change in meaning.^8^ The frequency and concreteness data covered 54% of the included terms in Study 2.^9^ Appendix E shows the comparison between different filtering methods for this rating dataset.^10^ *brm*(*ratings* ∼ 1 + *age group* * *pair type* * *changed* + (1 + *changed* * *pair type* | *subject*) + (1 + *age group* | *pair*), *family* = *cumulative*(“*logit*”)).^11^ See Appendix F for details concerning the model.^12^ *brm*(*ratings* ∼ *sim*_1940_ + *sim*_1950_ + *sim*_1960_ + *sim*_1970_ + *sim*_1980_ + *sim*_1990_ + (1|*subject*).^13^ See Appendix H for details.^14^ The full tables of the ELPD differences can be found in Supplementary Materials.^15^ Overall, participants indicated changed words as unfamiliar (≈25%) more than unchanged words (≈10%). The proportions were similar across age groups: YA indicated 25.3% of the changed words as unfamiliar, whereas OA indicated 24.3% as unfamiliar, and the unchanged word rates were respectively 9.5% and 12.1% for YA and OA. As a caveat, a few participants in both age groups indicated that they thought the “unfamiliar” indication was an attention check verifying whether they remembered any of the terms from the previous page. Because of this, these unfamiliarity ratings might potentially overestimate participants’ actual familiarity.

## Supplementary Material



## Figures and Tables

**Figure A1.**
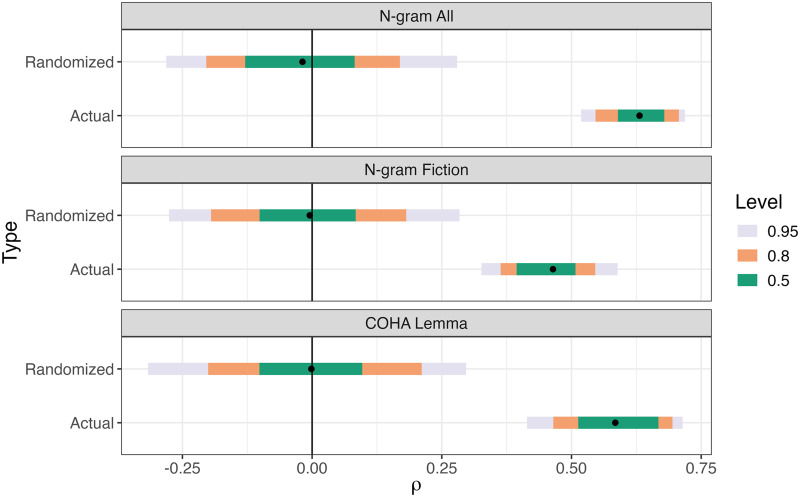
Distribution of correlations for both the actual correlations between decade-specific similarity values from Histwords and the versions with randomly shuffled decade labels.

**Figure A2.**
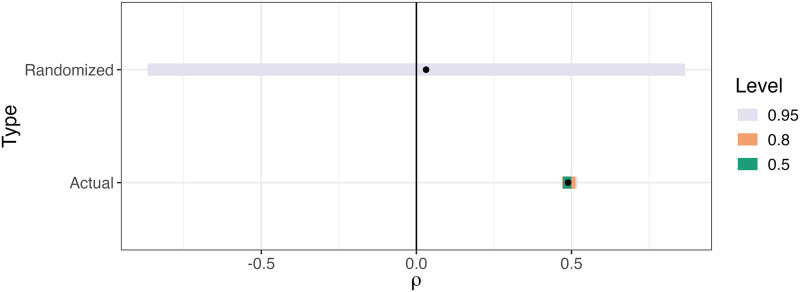
Distribution of correlations for both the actual correlations between age group-specific similarities from SWOW and the randomized permutations.

**Figure C1.**
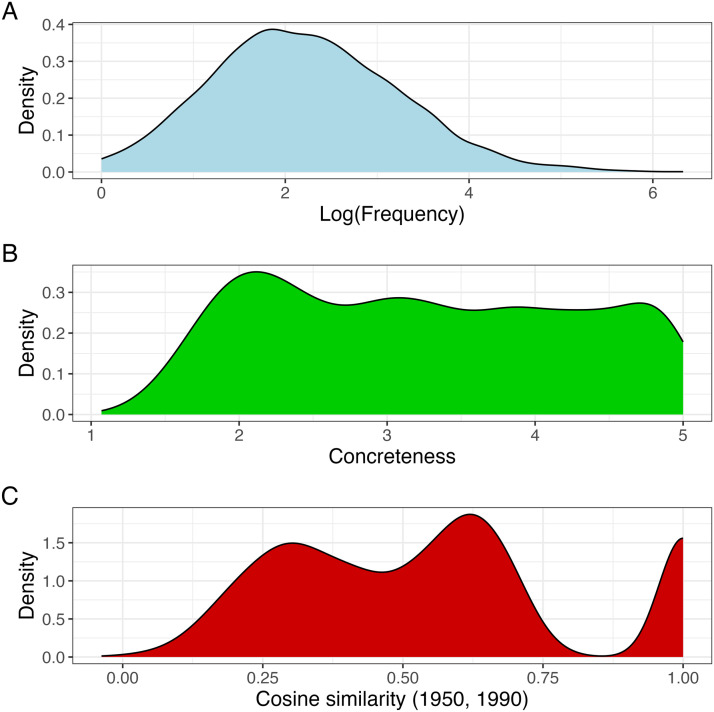
(A) Distribution of frequency based on SUBTLEX. (B) Term concreteness from Brysbaert, Warriner, et al. (2014). (C) Self-cosine similarity between the 1950s and 1990s, using HistWords.

**Figure D1.**
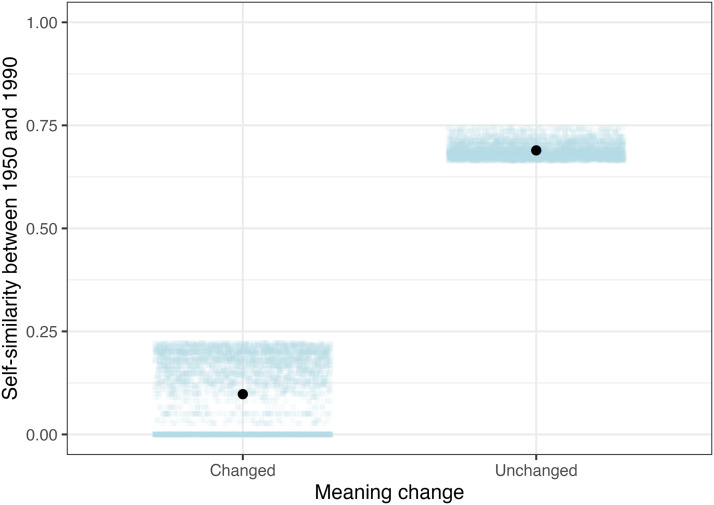
Cosine similarity for the changed and unchanged target words. Each light-blue point represents an individual target word, and the black points represent the averages per group.

**Figure E1.**
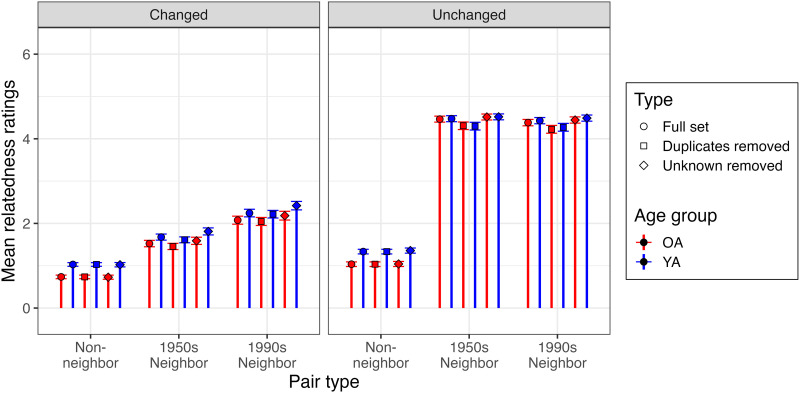
Mean relatedness ratings, from Study 2, by target word type (changed vs. unchanged in meaning from 1950 to 1990) and pair type. Error bars represent 95% confidence intervals over pair means. The three datasets are shown: full, duplicates removed, unknown removed.

**Figure F1.**
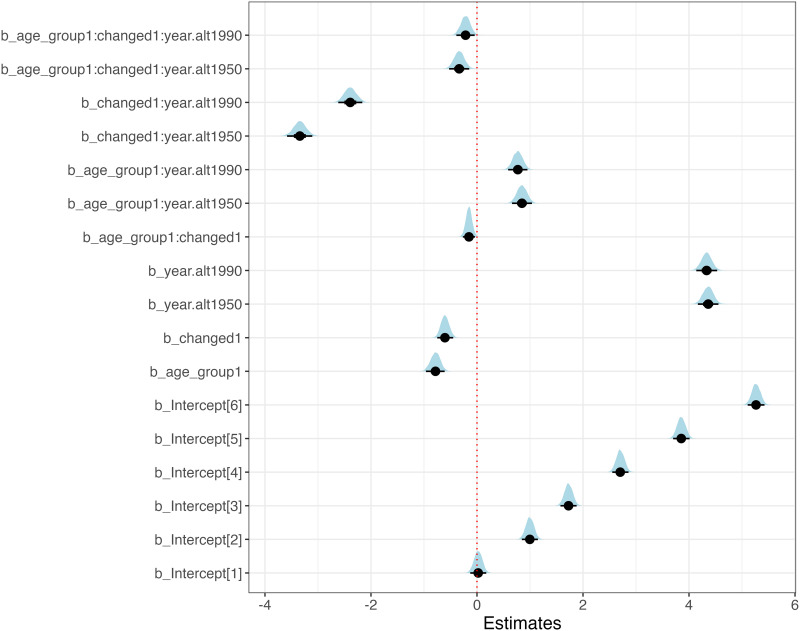
Model estimates from the Bayesian model. Point-ranges represent the average effect size and 95% CrI, and the densities represent the distribution of posterior draws.

**Figure F2.**
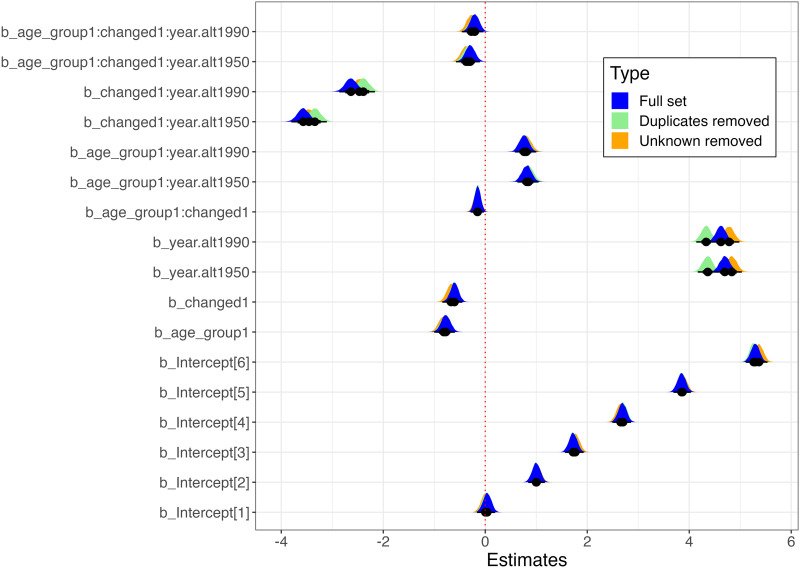
Model estimates from the same Bayesian model trained on different subsets of the data. Point-ranges represent the average effect size and 95% CrI, and the densities represent the distribution of posterior draws.

**Figure G1.**
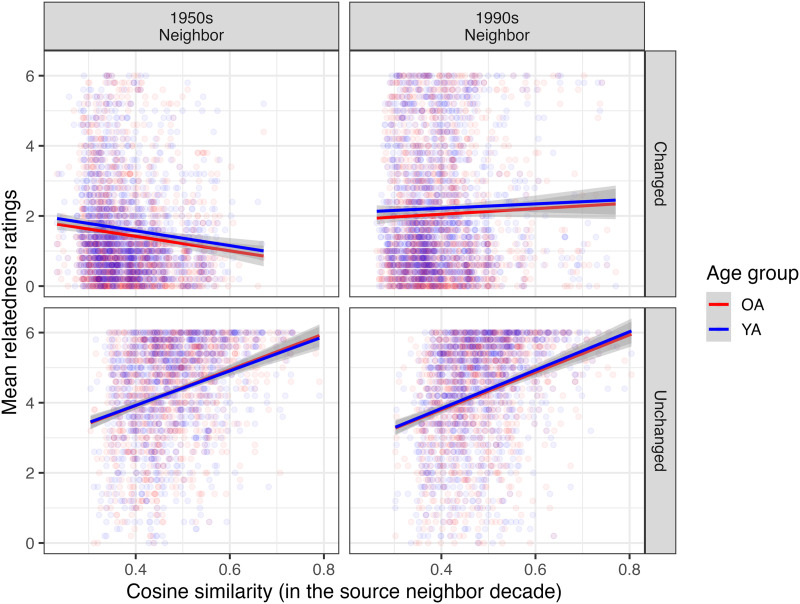
Scatter plot of relatedness ratings from both age groups against the cosine similarity between the word pairs from 1950s and 1990s embeddings from HistWords. The cosine similarity was calculated based on the decade of the neighbor source (i.e., cosine similarity based on the 1950s embeddings for the 1950s neighbor). The plot is split by neighbor type and meaning change. Each point represents a cue-neighbor pair, averaged across five ratings.

**Figure H1.**
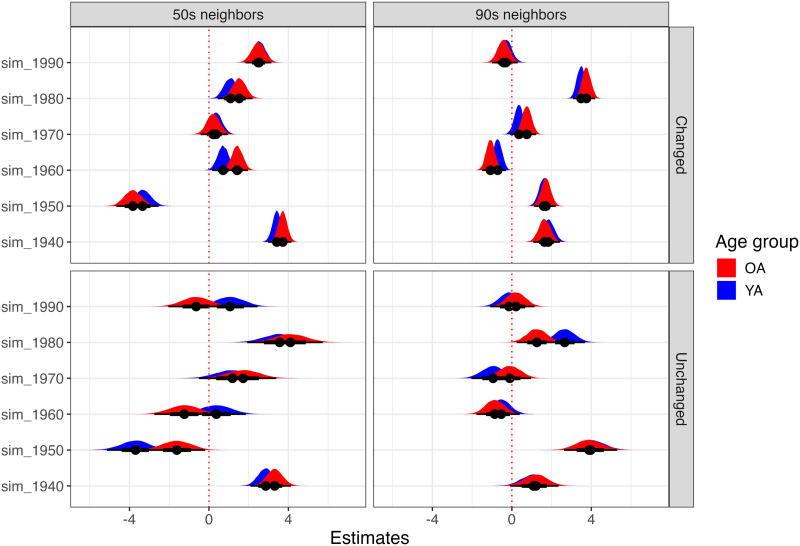
Posterior distributions of parameter estimates from the Bayesian models of relatedness ratings using N-gram All.

**Figure H2.**
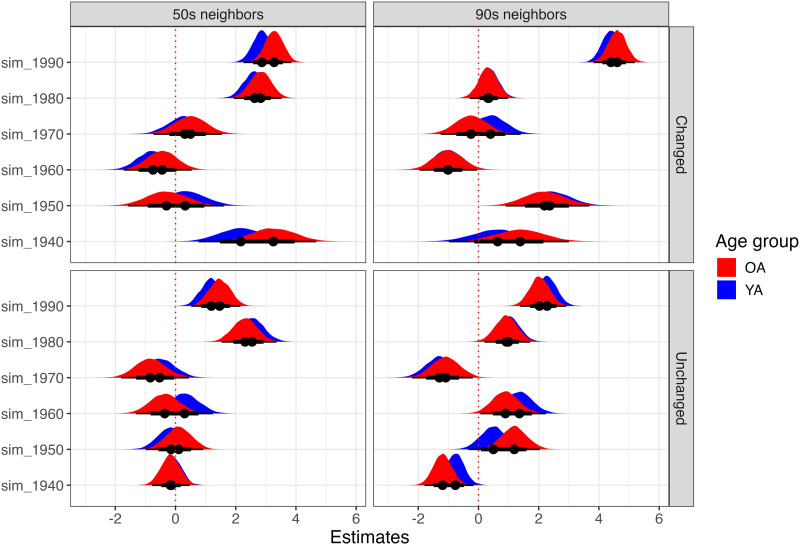
Posterior distributions of parameter estimates from the Bayesian models of relatedness ratings using N-gram Fiction.

**Figure H3.**
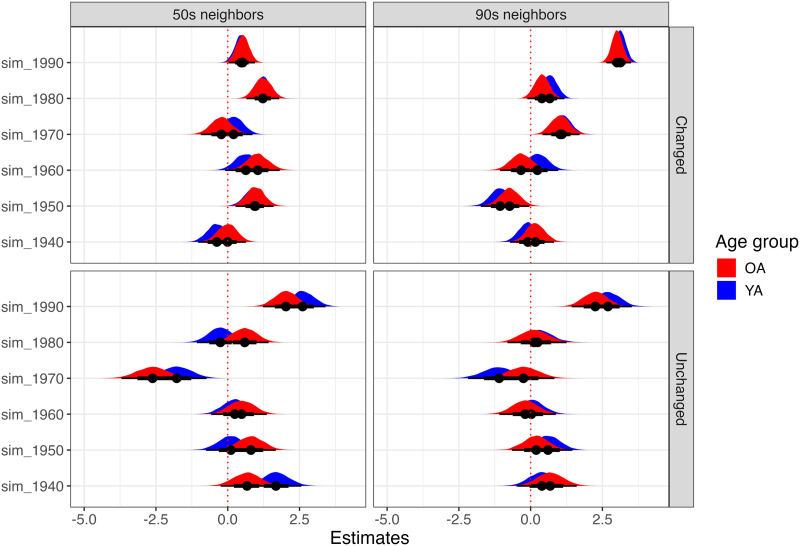
Posterior distributions of parameter estimates from the Bayesian models of relatedness ratings using COHA Lemma.

## APPENDIX A

### Study 1: Permutation Tests for Within Data Source RSA

We ran a permutation test to verify the robustness of the trend in meaning change over time in HistWords. We ran 500 iterations of random shuffling of the decade values when calculating the correlation between the decade difference (e.g., 10 years between 1980s and 1990s) and the correlation between the RSMs. The true correlations (*ρ*_ngram all_ = 0.63, *ρ*_ngram fiction_ = 0.46, *ρ*_coha lemma_ = 0.59) were on average much higher than the correlations on randomly shuffled were on average much higher than the correlations on randomly shuffled decades (*ρ*_ngram all_ = −0.02, *ρ*_ngram fiction_ = −0.005, *ρ*_coha lemma_ = −0.001), verifying the trend that decades that are closer together have more similar lexical semantics, as seen in Figure A1.

We ran a similar permutation test to verify whether lexico-semantic representations derived from word association data are more correlated for groups that are closer in age. To do so, we ran 500 iterations of random shuffling of the age cohort ranges when calculating the correlation between the age difference (e.g., 15 years between 20 years old and 35 years old) and the correlation between the RSMs. As seen in Figure A2, the true correlations had an average correlation of 0.49, which is higher than the randomized correlations (mean *ρ* = 0.03). However, this was within the 95% interval for the randomized correlations, confirming that the current data do not provide evidence that individuals closer in age have more similar lexical semantics.

## APPENDIX B

### Study 1: Ablation Analysis

All models were trained using a 5-fold cross-validation. The full model equation was:

*sim*_*swow*_ = *β*_0_ + (*β*_1_ * *sim*_1900_) + (*β*_2_ * *sim*_1910_) + (*β*_3_ * *sim*_1920_) + (*β*_4_ * *sim*_1930_) + (*β*_5_ * *sim*_1940_) + (*β*_6_ * *sim*_1950_) + (*β*_7_ * *sim*_1960_) + (*β*_8_ * *sim*_1970_) + (*β*_9_ * *sim*_1980_) + (*β*_10_ * *sim*_1990_) + *ε*

When a decade was ‘ablated’, it was removed from the full model equation. For example, ablating the 1910s would be done using the following equation:

*sim*_*swow*_ = *β*_0_ + (*β*_1_ * *sim*_1900_) + (*β*_2_ * *sim*_1920_) + (*β*_3_ * *sim*_1930_) + (*β*_4_ * *sim*_1940_) + (*β*_5_ * *sim*_1950_) + (*β*_6_ * *sim*_1960_) + (*β*_7_ * *sim*_1970_) + (*β*_8_ * *sim*_1980_) + (*β*_9_ * *sim*_1990_) + *ε*

## APPENDIX C

### Study 2: Stimulus Properties

As with Study 1, the frequency, concreteness, and self-meaning change between 1950s and 1990s for the stimuli used in Study 2 can be see in Figure C1.

## APPENDIX D

### Study 2: Quantifying Meaning Change in Stimuli

As mentioned in the main text, we selected two sets of target words that had undergone the most change in meaning and those that had generally remained unchanged. Figure D1 shows the distribution of similarity values for the changed and unchanged target words. The cosine similarity for each target word was calculated between the 1950s and 1990s.

Moreover, we also calculated the average similarity between a target word and the nearest neighbors. As seen in Figure D2, the changed target-neighbor pairs generally peak in similarity at the decade they were drawn from, whereas the unchanged target-neighbor pairs had consistent cosine similarity across decades.

## APPENDIX E

### Study 2: Data Subset Comparison

We compared the three different versions of the dataset: the full original dataset, one with the duplicates removed from the full set, and one with just the pairs rated as “known” from the original set. The majority of duplicate instances were 1–2 shared pairs between the 1950s and 1990s, with the unchanged more frequently having overlaps than the changed. In order to ensure that there would be enough pairs per cue, we evenly split the duplicate pairs between the 1950s and 1990s, such that if there were 2 shared pairs, for example, we would remove one from each. For the dataset with only known words, we excluded pairs if at least one of the words was marked as unknown. Upon visual inspection, there is very little difference between the three sets (Figure E1): mean relatedness ratings for the duplicates removed version are slightly below those for the full set, whereas ratings for the unknown removed are slightly above. The pattern across conditions and age groups appears the same across all three versions of the dataset.

## APPENDIX F

### Study 2: Main Statistical Model

As stated in the main text, we used a Bayesian multilevel ordinal model to test the relationship between age group, pair types (1990s, 1950s, or non-neighbor), meaning change, their interactions, and the average relatedness ratings for each target-neighbor pair. The YA group, unchanged target words, and non-neighbor pairs were coded as the reference levels for the corresponding variables. We used the default, uninformative priors and trained the model on the ‘duplicates removed’ subset of the data.

Figure F1 shows the the posterior distribution for each fixed parameter from the main Bayesian model.

We ran three iterations of this model on the three different versions of the dataset (full set, duplicates removed, familiar pairs only, see Appendix E). As seen in Figure F2, there was very little difference between the model fit between the three subsets, with the exception of the coefficients for the pair type (1950s, 1990s). This difference is most likely due to those having a slight difference in average ratings (Figure E1).

## APPENDIX G

### Study 2: Visualizing the Correlation Between Ratings and Embeddings

Figure G1 shows scatterplots comparing the relatedness judgments for 1950s and 1990s neighbor pairs to the cosine similarity values from embeddings from the source decade (1950s and 1990s respectively). For the unchanged target words, the relatedness judgments are positively correlated with the cosine similarity values (*ρ*_1950*s*_,*unchanged* ≈ 0.3, *ρ*_1990*s*_,*unchanged* ≈ 0.32). However, for the changed words, there does not seem to be a strong relationship between the 1990s cosine similarity values and the relatedness ratings (*ρ* ≈ 0.03), whereas the 1950s neighbor ratings are inversely related (*ρ* ≈ −0.14) to the cosine similarity values from the 1950s embeddings. This may be partially due to the fact that pairs containing target words that changed in meaning were rated as less related overall. It is noteworthy that the correlations do not appear to differ between age groups (Mean difference in ratings = 0.0072).

## APPENDIX H

### Study 2: Ablation Analysis

As stated in the main text, we trained Bayesian multilevel ordinal models to predict participant relatedness ratings as a combination of decade-level similarity from HistWords, and ablated individual decades to quantify their impact via model comparison. We separately trained models for the two types of meaning change, since the range of similarity values greatly differed between the two (Changed: *0* < *cosine similarity* < *0.25*, Unchanged: *0.65* < *cosine similarity* < *0.75*). Likewise, we separately trained models for the younger and older adults’ ratings for the two different pair types. This resulted in 56 models total (7 ablations × 2 age groups × 2 neighbor types × 2 meaning change types).

The full formula was as follows:

*brm*(*ratings* ∼ *sim*_1940_ + *sim*_1950_ + *sim*_1960_ + *sim*_1970_ + *sim*_1980_ + *sim*_1990_ + (1|*subject*)

where *ratings* are the relatedness ratings from one of the age groups, and *sim*_*year*_ is the similarity between the word pair based on a decade-specific embedding from HistWords. Figure H1 shows the posterior distributions of parameter estimates for only the **full** models using N-gram All, Figure H2 for N-gram Fiction, and Figure H3 for COHA Lemma.

As with >Study 1, when a decade was ‘ablated’, it was removed from the full model equation. Ablating the 1960s would be done using the following equation:

*brm*(*ratings* ∼ *sim*_1940_ + *sim*_1950_ + *sim*_1970_ + *sim*_1980_ + *sim*_1990_ + (1|*subject*).

To quantify the ablation impact of each predictor, we we used the loo_compare() function in R to calculate the expected log predictive density difference between each ablated model and the full model.

## References

[bib1] Abbott, J. T., Austerweil, J. L., & Griffiths, T. L. (2012). Human memory search as a random walk in a semantic network. In F. Pereira, C. J. C. Burges, L. Bottou, & K. Q. Weinberger (Eds.), Proceedings of the 26th International Conference on Neural Information Processing Systems (Vol. 2, pp. 3041–3049). Curran Associates, Inc.

[bib2] Baayen, R. H., Tomaschek, F., Gahl, S., & Ramscar, M. (2017). The ecclesiastes principle in language change. In I. M. Hundt, S. Mollin, & S. E. Pfenninger (Eds.), The changing English language: Psycholinguistic perspectives (pp. 21–48). Cambridge University Press. 10.1017/9781316091746.002

[bib3] Brysbaert, M., & New, B. (2009). Moving beyond Kučera and Francis: A critical evaluation of current word frequency norms and the introduction of a new and improved word frequency measure for American English. Behavior Research Methods, 41(4), 977–990. 10.3758/BRM.41.4.977, 19897807

[bib4] Brysbaert, M., Stevens, M., De Deyne, S., Voorspoels, W., & Storms, G. (2014). Norms of age of acquisition and concreteness for 30,000 Dutch words. Acta Psychologica, 150, 80–84. 10.1016/j.actpsy.2014.04.010, 24831463

[bib5] Brysbaert, M., Stevens, M., Mandera, P., & Keuleers, E. (2016). How many words do we know? Practical estimates of vocabulary size dependent on word definition, the degree of language input and the participant’s age. Frontiers in Psychology, 7, 1116. 10.3389/fpsyg.2016.01116, 27524974 PMC4965448

[bib6] Brysbaert, M., Warriner, A. B., & Kuperman, V. (2014). Concreteness ratings for 40 thousand generally known English word lemmas. Behavior Research Methods, 46(3), 904–911. 10.3758/s13428-013-0403-5, 24142837

[bib7] Bürkner, P.-C. (2017). brms: An R package for Bayesian multilevel models using Stan. Journal of Statistical Software, 80(1), 1–28. 10.18637/jss.v080.i01

[bib8] Bybee, J. (2015). Language change. Cambridge University Press. 10.1017/CBO9781139096768

[bib9] Bynon, T. (1977). Historical linguistics. Cambridge University Press. 10.1017/CBO9781139165709

[bib10] Castro, N., Curley, T., & Hertzog, C. (2021). Category norms with a cross-sectional sample of adults in the United States: Consideration of cohort, age, and historical effects on semantic categories. Behavior Research Methods, 53(2), 898–917. 10.3758/s13428-020-01454-9, 32901344 PMC7937767

[bib11] Chater, N., & Christiansen, M. H. (2010). Language acquisition meets language evolution. Cognitive Science, 34(7), 1131–1157. 10.1111/j.1551-6709.2009.01049.x, 21564247

[bib12] Cosgrove, A. L., Kenett, Y. N., Beaty, R. E., & Diaz, M. T. (2021). Quantifying flexibility in thought: The resiliency of semantic networks differs across the lifespan. Cognition, 211, 104631. 10.1016/j.cognition.2021.104631, 33639378 PMC8058279

[bib13] Davies, M. (2012). Expanding horizons in historical linguistics with the 400-million word Corpus of Historical American English. Corpora, 7(2), 121–157. 10.3366/cor.2012.0024

[bib14] De Deyne, S., Navarro, D. J., Perfors, A., Brysbaert, M., & Storms, G. (2019). The “Small World of Words” English word association norms for over 12,000 cue words. Behavior Research Methods, 51(3), 987–1006. 10.3758/s13428-018-1115-7, 30298265

[bib15] De Deyne, S., Navarro, D. J., Perfors, A., & Storms, G. (2016). Structure at every scale: A semantic network account of the similarities between unrelated concepts. Journal of Experimental Psychology: General, 145(9), 1228–1254. 10.1037/xge0000192, 27560855

[bib16] Devlin, J., Chang, M.-W., Lee, K., & Toutanova, K. (2019). BERT: Pre-training of deep bidirectional transformers for language understanding. arXiv. 10.48550/arXiv.1810.04805

[bib17] Dubossarsky, H., De Deyne, S., & Hills, T. T. (2017). Quantifying the structure of free association networks across the life span. Developmental Psychology, 53(8), 1560–1570. 10.1037/dev0000347, 28569517

[bib18] Elman, J. L. (2009). On the meaning of words and dinosaur bones: Lexical knowledge without a lexicon. Cognitive Science, 33(4), 547–582. 10.1111/j.1551-6709.2009.01023.x, 19662108 PMC2721468

[bib19] Ettinger, A., & Linzen, T. (2016). Evaluating vector space models using human semantic priming results. In Proceedings of the 1st Workshop on Evaluating Vector-Space Representations for NLP (pp. 72–77). Association for Computational Linguistics. 10.18653/v1/W16-2513

[bib20] Firth, J. R. (1957). A synopsis of linguistic theory, 1930–1955. In Studies in linguistic analysis (pp. 1–32). Blackwell.

[bib21] Fitneva, S. A., & Christiansen, M. H. (2011). Looking in the wrong direction correlates with more accurate word learning. Cognitive Science, 35(2), 367–380. 10.1111/j.1551-6709.2010.01156.x, 21429004

[bib22] Five Graces Group, Beckner, C., Blythe, R., Bybee, J., Christiansen, M. H., Croft, W., Ellis, N. C., Holland, J., Ke, J., Larsen-Freeman, D., & Schoenemann, T. (2009). Language is a complex adaptive system: Position paper. Language Learning, 59(S1), 1–26. 10.1111/j.1467-9922.2009.00533.x

[bib23] Gerz, D., Vulić, I., Hill, F., Reichart, R., & Korhonen, A. (2016). SimVerb-3500: A large-scale evaluation set of verb similarity. In J. Su, K. Duh, & X. Carreras (Eds.), Proceedings of the 2016 Conference on Empirical Methods in Natural Language Processing (pp. 2173–2182). Association for Computational Linguistics. 10.18653/v1/D16-1235

[bib24] Goldstone, R. (1994). An efficient method for obtaining similarity data. Behavior Research Methods, Instruments, & Computers, 26(4), 381–386. 10.3758/BF03204653

[bib25] Goldstone, R. L., Medin, D. L., & Halberstadt, J. (1997). Similarity in context. Memory & Cognition, 25(2), 237–255. 10.3758/BF03201115, 9099074

[bib26] Grand, G., Blank, I. A., Pereira, F., & Fedorenko, E. (2022). Semantic projection recovers rich human knowledge of multiple object features from word embeddings. Nature Human Behaviour, 6(7), 975–987. 10.1038/s41562-022-01316-8, 35422527 PMC10349641

[bib27] Hamilton, W. L., Leskovec, J., & Jurafsky, D. (2018). Diachronic word embeddings reveal statistical laws of semantic change. arXiv. 10.48550/arXiv.1605.09096

[bib28] Harris, Z. S. (1954). Distributional structure. Word, 10(2–3), 146–162. 10.1080/00437956.1954.11659520

[bib29] Hartshorne, J. K., & Germine, L. T. (2015). When does cognitive functioning peak? The asynchronous rise and fall of different cognitive abilities across the life span. Psychological Science, 26(4), 433–443. 10.1177/0956797614567339, 25770099 PMC4441622

[bib30] Hill, F., Reichart, R., & Korhonen, A. (2015). SimLex-999: Evaluating semantic models with (genuine) similarity estimation. Computational Linguistics, 41(4), 665–695. 10.1162/COLI_a_00237

[bib31] Hills, T. T., Jones, M. N., & Todd, P. M. (2012). Optimal foraging in semantic memory. Psychological Review, 119(2), 431–440. 10.1037/a0027373, 22329683

[bib32] Hills, T. T., Mata, R., Wilke, A., & Samanez-Larkin, G. R. (2013). Mechanisms of age-related decline in memory search across the adult life span. Developmental Psychology, 49(12), 2396–2404. 10.1037/a0032272, 23586941 PMC3842414

[bib33] Hills, T. T., Todd, P. M., & Jones, M. N. (2015). Foraging in semantic fields: How we search through memory. Topics in Cognitive Science, 7(3), 513–534. 10.1111/tops.12151, 26097107

[bib34] Hilpert, M., & Gries, S. T. (2009). Assessing frequency changes in multistage diachronic corpora: Applications for historical corpus linguistics and the study of language acquisition. Literary and Linguistic Computing, 24(4), 385–401. 10.1093/llc/fqn012

[bib35] Johns, B. T., & Jones, M. N. (2022). Content matters: Measures of contextual diversity must consider semantic content. Journal of Memory and Language, 123, 104313. 10.1016/j.jml.2021.104313

[bib36] Kamath, G., Yang, M., Reddy, S., Sonderegger, M., & Card, D. (2025). Semantic change in adults is not primarily a generational phenomenon. Proceedings of the National Academy of Sciences, 122(31), e2426815122. 10.1073/pnas.2426815122, 40720652 PMC12337318

[bib37] Kriegeskorte, N., Mur, M., & Bandettini, P. (2008). Representational similarity analysis—Connecting the branches of systems neuroscience. Frontiers in Systems Neuroscience, 2, 4. 10.3389/neuro.06.004.2008, 19104670 PMC2605405

[bib38] Kumar, A. A., Steyvers, M., & Balota, D. A. (2022). A critical review of network-based and distributional approaches to semantic memory structure and processes. Topics in Cognitive Science, 14(1), 54–77. 10.1111/tops.12548, 34092042

[bib39] Kuperman, V., Stadthagen-Gonzalez, H., & Brysbaert, M. (2012). Age-of-acquisition ratings for 30,000 English words. Behavior Research Methods, 44(4), 978–990. 10.3758/s13428-012-0210-4, 22581493

[bib40] Lenci, A. (2018). Distributional models of word meaning. Annual Review of Linguistics, 4, 151–171. 10.1146/annurev-linguistics-030514-125254

[bib41] Lenth, R. V. (2024). emmeans: Estimated marginal means, aka least-squares means [Computer software manual]. https://rvlenth.github.io/emmeans/

[bib42] Lewis, M., Zettersten, M., & Lupyan, G. (2019). Distributional semantics as a source of visual knowledge. Proceedings of the National Academy of Sciences, 116(39), 19237–19238. 10.1073/pnas.1910148116, 31488726 PMC6765286

[bib43] Li, Y., & Siew, C. S. Q. (2022). Diachronic semantic change in language is constrained by how people use and learn language. Memory & Cognition, 50(6), 1284–1298. 10.3758/s13421-022-01331-0, 35767153 PMC9365724

[bib44] Maciejewski, G., Rodd, J. M., Mon-Williams, M., & Klepousniotou, E. (2020). The cost of learning new meanings for familiar words. Language, Cognition and Neuroscience, 35(2), 188–210. 10.1080/23273798.2019.1642500

[bib45] McRae, K., Khalkhali, S., & Hare, M. (2012). Semantic and associative relations in adolescents and young adults: Examining a tenuous dichotomy. In V. F. Reyna, S. B. Chapman, M. R. Dougherty, & J. Confrey (Eds.), The adolescent brain: Learning, reasoning, and decision making (pp. 39–66). American Psychological Association. 10.1037/13493-002

[bib46] Michel, J.-B., Shen, Y. K., Aiden, A. P., Veres, A., Gray, M. K., The Google Books Team, Pickett, J. P., Hoiberg, D., Clancy, D., Norvig, P., Orwant, J., Pinker, S., Nowak, M. A., & Aiden, E. L. (2011). Quantitative analysis of culture using millions of digitized books. Science, 331(6014), 176–182. 10.1126/science.1199644, 21163965 PMC3279742

[bib47] Mikolov, T., Chen, K., Corrado, G., & Dean, J. (2013). Efficient estimation of word representations in vector space. arXiv. 10.48550/arXiv.1301.3781

[bib48] Pechenick, E. A., Danforth, C. M., & Dodds, P. S. (2015). Characterizing the Google Books Corpus: Strong limits to inferences of socio-cultural and linguistic evolution. PLOS ONE, 10(10), e0137041. 10.1371/journal.pone.0137041, 26445406 PMC4596490

[bib49] Periti, F., & Tahmasebi, N. (2024). A systematic comparison of contextualized word embeddings for lexical semantic change. In K. Duh, H. Gomez, & S. Bethard (Eds.), Proceedings of the 2024 Conference of the North American Chapter of the Association for Computational Linguistics: Human Language Technologies (Volume 1: Long Papers) (pp. 4262–4282). Association for Computational Linguistics. 10.18653/v1/2024.naacl-long.240

[bib50] Piantadosi, S. T. (2014). Zipf’s word frequency law in natural language: A critical review and future directions. Psychonomic Bulletin & Review, 21(5), 1112–1130. 10.3758/s13423-014-0585-6, 24664880 PMC4176592

[bib51] Qiu, W., & Xu, Y. (2022). HistBERT: A pre-trained language model for diachronic lexical semantic analysis. arXiv. 10.48550/arXiv.2202.03612

[bib52] Ramiro, C., Srinivasan, M., Malt, B. C., & Xu, Y. (2018). Algorithms in the historical emergence of word senses. Proceedings of the National Academy of Sciences, 115(10), 2323–2328. 10.1073/pnas.1714730115, 29463738 PMC5877971

[bib53] Ramscar, M., Hendrix, P., Shaoul, C., Milin, P., & Baayen, H. (2014). The myth of cognitive decline: Non-linear dynamics of lifelong learning. Topics in Cognitive Science, 6(1), 5–42. 10.1111/tops.12078, 24421073

[bib54] Reilly, J., Shain, C., Borghesani, V., Kuhnke, P., Vigliocco, G., Peelle, J. E., Mahon, B. Z., Buxbaum, L. J., Majid, A., Brysbaert, M., Borghi, A. M., De Deyne, S., Dove, G., Papeo, L., Pexman, P. M., Poeppel, D., Lupyan, G., Boggio, P., Hickok, G., … Vinson, D. (2025). What we mean when we say semantic: Toward a multidisciplinary semantic glossary. Psychonomic Bulletin & Review, 32(1), 243–280. 10.3758/s13423-024-02556-7, 39231896 PMC11836185

[bib55] Ritchie, J. B., Bracci, S., & Op de Beeck, H. (2017). Avoiding illusory effects in representational similarity analysis: What (not) to do with the diagonal. NeuroImage, 148, 197–200. 10.1016/j.neuroimage.2016.12.079, 28069538

[bib56] Rodd, J. M. (2020). Settling into semantic space: An ambiguity-focused account of word-meaning access. Perspectives on Psychological Science, 15(2), 411–427. 10.1177/1745691619885860, 31961780

[bib57] Rodd, J. M., Cai, Z. G., Betts, H. N., Hanby, B., Hutchinson, C., & Adler, A. (2016). The impact of recent and long-term experience on access to word meanings: Evidence from large-scale internet-based experiments. Journal of Memory and Language, 87, 16–37. 10.1016/j.jml.2015.10.006

[bib58] Ryskin, R. A., Qi, Z., Duff, M. C., & Brown-Schmidt, S. (2017). Verb biases are shaped through lifelong learning. Journal of Experimental Psychology: Learning, Memory, and Cognition, 43(5), 781–794. 10.1037/xlm0000341, 27762578 PMC5398958

[bib59] Siew, C. S. Q., Wulff, D. U., Beckage, N. M., & Kenett, Y. N. (2019). Cognitive network science: A review of research on cognition through the lens of network representations, processes, and dynamics. Complexity, 2019, 2108423. 10.1155/2019/2108423

[bib60] Sivula, T., Magnusson, M., Matamoros, A. A., & Vehtari, A. (2020). Uncertainty in Bayesian leave-one-out cross-validation based model comparison. arXiv. 10.48550/arXiv.2008.10296

[bib61] Smith, K., Smith, A. D. M., & Blythe, R. A. (2011). Cross-situational learning: An experimental study of word-learning mechanisms. Cognitive Science, 35(3), 480–498. 10.1111/j.1551-6709.2010.01158.x

[bib62] Srinivasan, M., & Rabagliati, H. (2015). How concepts and conventions structure the lexicon: Cross-linguistic evidence from polysemy. Lingua, 157, 124–152. 10.1016/j.lingua.2014.12.004

[bib63] Wittgenstein, L. (1953). Philosophical investigations. Macmillan.

[bib64] Xu, Y., & Kemp, C. (2015). A computational evaluation of two laws of semantic change. In D. C. Noelle, R. Dale, A. S. Warlaumont, J. Yoshimi, T. Matlock, C. D. Jennings, & P. P. Maglio (Eds.), Proceedings of the 37th Annual Conference of the Cognitive Science Society (pp. 2703–2708). Cognitive Science Society.

[bib65] Yu, C., & Smith, L. B. (2007). Rapid word learning under uncertainty via cross-situational statistics. Psychological Science, 18(5), 414–420. 10.1111/j.1467-9280.2007.01915.x, 17576281

[bib66] Zipf, G. (1935). The psycho-biology of language: An introduction to dynamic philology. Houghton Mifflin.

