# Supplementary material for: Semantic Representations Are Updated Across the Lifespan Reflecting Diachronic Language Change
Source: Open Mind (Camb). 2025 Dec 18;9:2114–48. doi: 10.1162/OPMI.a.315 (PMC12768554; doi:10.1162/OPMI.a.315)
Supplement: Supplementary file 1 [file opmi-09-2114-s001.pdf]

Semantic representations are updated across the lifespan reflecting diachronic language change

Ellis Cain & Rachel Ryskin
University of California, Merced

Supplemental Material

Study 1: Ablation F-test results

The following tables show the numeric impact of ablation on model performance ($full\ model - ablated\ model$) for AIC, log-likelihood, and R^2. For N-gram All, see Tables 1, 2, 3; for N-gram Fiction, see Tables 4, 5, 6; for COHA Lemma, see Tables 7, 8, 9.

As mentioned in the main text, F-tests comparing the model fit of the full and ablated models showed that the full model always predicts the association-based similarity values significantly better than the ablated models ($ps < 0.001$). The F-test results for each comparison can be found in Tables 10 through 18.

Study 2: Ablated model comparisons

Tables 19, 20 show the results for N-gram All, Tables 21, 22 for N-gram Fiction, and Tables 23, 24for COHA Lemma.

Cohort	1900	1910	1920	1930	1940
20-35	-5.29e+04	-1.21e+04	-3.09e+03	-2.78e+03	-3.49e+03
35-50	-5.01e+04	-1.17e+04	-3.68e+03	-3.06e+03	-3.67e+03
50-90	-4.55e+04	-1.13e+04	-3.06e+03	-2.73e+03	-3.45e+03
Cohort	1950	1960	1970	1980	1990
20-35	-5.49e+02	-2.24e+03	-1.07e+04	-3.83e+04	-1.38e+05
35-50	-5.23e+02	-1.86e+03	-8.43e+03	-3.12e+04	-1.15e+05
50-90	-3.16e+02	-1.58e+03	-6.10e+03	-2.39e+04	-8.83e+04

Table 1

Ablation impact on model performance for N-gram All, for AIC. More negative numbers indicate a larger ablation impact.

Cohort	1900	1910	1920	1930	1940
20-35	2.64e+04	6.06e+03	1.55e+03	1.39e+03	1.75e+03
35-50	2.50e+04	5.85e+03	1.84e+03	1.53e+03	1.84e+03
50-90	2.27e+04	5.65e+03	1.53e+03	1.37e+03	1.72e+03
Cohort	1950	1960	1970	1980	1990
20-35	2.75e+02	1.12e+03	5.34e+03	1.91e+04	6.88e+04
35-50	2.63e+02	9.31e+02	4.21e+03	1.56e+04	5.75e+04
50-90	1.59e+02	7.89e+02	3.05e+03	1.19e+04	4.42e+04

Table 2

Ablation impact on model performance for N-gram All, for log-likelihood. Larger numbers indicate a larger ablation impact.

Cohort	1900	1910	1920	1930	1940
20-35	1.98e-03	4.54e-04	1.16e-04	1.04e-04	1.31e-04
35-50	1.93e-03	4.50e-04	1.42e-04	1.18e-04	1.41e-04
50-90	1.85e-03	4.60e-04	1.25e-04	1.11e-04	1.40e-04
Cohort	1950	1960	1970	1980	1990
20-35	2.06e-05	8.38e-05	4.00e-04	1.43e-03	5.17e-03
35-50	2.02e-05	7.16e-05	3.24e-04	1.20e-03	4.43e-03
50-90	1.30e-05	6.42e-05	2.48e-04	9.72e-04	3.60e-03

Table 3

Ablation impact on model performance for N-gram All, for R^2. Larger numbers indicate a larger ablation impact.

Cohort	1900	1910	1920	1930	1940
20-35	-6.24e+03	-5.58e+03	-1.46e+03	-2.61e+00	-1.29e+01
35-50	-7.44e+03	-5.65e+03	-1.92e+03	1.98e+00	1.70e+00
50-90	-6.98e+03	-4.92e+03	-2.17e+03	-3.35e+00	-4.03e+00
Cohort	1950	1960	1970	1980	1990
20-35	-7.88e+02	-1.11e+04	-7.77e+03	-9.42e+04	-2.19e+05
35-50	-8.27e+02	-1.04e+04	-6.93e+03	-8.42e+04	-1.99e+05
50-90	-9.23e+02	-9.58e+03	-6.09e+03	-7.15e+04	-1.57e+05

Table 4

Ablation impact on model performance for N-gram Fiction, for AIC. More negative numbers indicate a larger ablation impact.

Cohort	1900	1910	1920	1930	1940
20-35	3.12e+03	2.79e+03	7.32e+02	2.30e+00	7.47e+00
35-50	3.72e+03	2.83e+03	9.62e+02	1.12e-02	1.50e-01
50-90	3.49e+03	2.46e+03	1.08e+03	2.67e+00	3.01e+00
Cohort	1950	1960	1970	1980	1990
20-35	3.95e+02	5.57e+03	3.89e+03	4.71e+04	1.09e+05
35-50	4.15e+02	5.20e+03	3.47e+03	4.21e+04	9.97e+04
50-90	4.63e+02	4.79e+03	3.05e+03	3.57e+04	7.86e+04

Table 5

Ablation impact on model performance for N-gram Fiction, for log-likelihood. Larger numbers indicate a larger ablation impact.

Cohort	1900	1910	1920	1930	1940
20-35	2.52e-04	2.25e-04	5.91e-05	1.86e-07	6.03e-07
35-50	3.06e-04	2.33e-04	7.92e-05	9.24e-10	1.23e-08
50-90	3.01e-04	2.12e-04	9.35e-05	2.30e-07	2.60e-07
Cohort	1950	1960	1970	1980	1990
20-35	3.19e-05	4.50e-04	3.14e-04	3.81e-03	8.87e-03
35-50	3.41e-05	4.28e-04	2.85e-04	3.47e-03	8.23e-03
50-90	3.98e-05	4.13e-04	2.62e-04	3.08e-03	6.79e-03

Table 6

Ablation impact on model performance for N-gram Fiction, for R^2. Larger numbers indicate a larger ablation impact.

Cohort	1900	1910	1920	1930	1940
20-35	-8.42e+03	-3.46e+03	-3.06e+03	-2.63e+02	-2.02e+03
35-50	-8.80e+03	-3.07e+03	-2.46e+03	-3.23e+02	-2.09e+03
50-90	-7.84e+03	-2.66e+03	-2.26e+03	-2.02e+02	-2.09e+03
Cohort	1950	1960	1970	1980	1990
20-35	-1.10e+03	-1.12e+03	-3.15e+01	-8.15e+02	-4.67e+04
35-50	-7.32e+02	-9.67e+02	-1.88e+02	-6.95e+02	-3.88e+04
50-90	-6.60e+02	-4.46e+02	-1.13e+02	-6.06e+02	-3.01e+04

Table 7

Ablation impact on model performance for COHA Lemma, for AIC. More negative numbers indicate a larger ablation impact.

Cohort	1900	1910	1920	1930	1940
20-35	4.21e+03	1.73e+03	1.53e+03	1.33e+02	1.01e+03
35-50	4.40e+03	1.53e+03	1.23e+03	1.62e+02	1.05e+03
50-90	3.92e+03	1.33e+03	1.13e+03	1.02e+02	1.05e+03
Cohort	1950	1960	1970	1980	1990
20-35	5.51e+02	5.61e+02	1.67e+01	4.08e+02	2.33e+04
35-50	3.67e+02	4.84e+02	9.51e+01	3.48e+02	1.94e+04
50-90	3.31e+02	2.24e+02	5.74e+01	3.04e+02	1.50e+04

Table 8

Ablation impact on model performance for COHA Lemma, for log-likelihood. Larger numbers indicate a larger ablation impact.

Cohort	1900	1910	1920	1930	1940
20-35	4.16e-04	1.71e-04	1.51e-04	1.31e-05	1.00e-04
35-50	4.43e-04	1.54e-04	1.24e-04	1.63e-05	1.05e-04
50-90	4.11e-04	1.40e-04	1.19e-04	1.07e-05	1.10e-04
Cohort	1950	1960	1970	1980	1990
20-35	5.45e-05	5.55e-05	1.65e-06	4.04e-05	2.31e-03
35-50	3.69e-05	4.87e-05	9.56e-06	3.50e-05	1.95e-03
50-90	3.47e-05	2.35e-05	6.02e-06	3.19e-05	1.58e-03

Table 9

Ablation impact on model performance for COHA Lemma, for R^2. Larger numbers indicate a larger ablation impact.

Ablation	F-test Result
-1900	$F(1, 23393673) = 52958.93$, $p < .001$
-1910	$F(1, 23393673) = 12121.44$, $p < .001$
-1920	$F(1, 23393673) = 3090.63$, $p < .001$
-1930	$F(1, 23393673) = 2779.51$, $p < .001$
-1940	$F(1, 23393673) = 3493.23$, $p < .001$
-1950	$F(1, 23393673) = 550.86$, $p < .001$
-1960	$F(1, 23393673) = 2237.47$, $p < .001$
-1970	$F(1, 23393673) = 10681.68$, $p < .001$
-1980	$F(1, 23393673) = 38291.93$, $p < .001$
-1990	$F(1, 23393673) = 137949.48$, $p < .001$

Table 10

F-test results for ablation models using N-gram All, with the 20–35 y.o. data. F-tests compare each ablated model against the full model.

Ablation	F-test Result
-1900	$F(1, 23032644) = 50128.18$, $p < .001$
-1910	$F(1, 23032644) = 11708.44$, $p < .001$
-1920	$F(1, 23032644) = 3679.54$, $p < .001$
-1930	$F(1, 23032644) = 3063.90$, $p < .001$
-1940	$F(1, 23032644) = 3675.20$, $p < .001$
-1950	$F(1, 23032644) = 525.40$, $p < .001$
-1960	$F(1, 23032644) = 1862.66$, $p < .001$
-1970	$F(1, 23032644) = 8430.05$, $p < .001$
-1980	$F(1, 23032644) = 31177.68$, $p < .001$
-1990	$F(1, 23032644) = 115297.98$, $p < .001$

Table 11

F-test results for ablation models using N-gram All, with the 35–50 y.o. data. F-tests compare each ablated model against the full model.

Ablation	F-test Result
-1900	F(1, 22085166) = 45534.65, p < .001
-1910	F(1, 22085166) = 11306.53, p < .001
-1920	F(1, 22085166) = 3064.15, p < .001
-1930	F(1, 22085166) = 2731.01, p < .001
-1940	F(1, 22085166) = 3449.89, p < .001
-1950	F(1, 22085166) = 318.33, p < .001
-1960	F(1, 22085166) = 1577.21, p < .001
-1970	F(1, 22085166) = 6100.72, p < .001
-1980	F(1, 22085166) = 23892.92, p < .001
-1990	F(1, 22085166) = 88498.66, p < .001

Table 12

F-test results for ablation models using N-gram All, with the 50–90 y.o. data. F-tests compare each ablated model against the full model.

Ablation	F-test Result
-1900	F(1, 22579927) = 6243.89, p < .001
-1910	F(1, 22579927) = 5580.79, p < .001
-1920	F(1, 22579927) = 1463.20, p < .001
-1930	F(1, 22579927) = 4.61, p = 0.03
-1940	F(1, 22579927) = 14.94, p < .001
-1950	F(1, 22579927) = 790.47, p < .001
-1960	F(1, 22579927) = 11141.57, p < .001
-1970	F(1, 22579927) = 7777.56, p < .001
-1980	F(1, 22579927) = 94349.14, p < .001
-1990	F(1, 22579927) = 219630.66, p < .001

Table 13

F-test results for ablation models using N-gram Fiction, with the 20–35 y.o. data. F-tests compare each ablated model against the full model.

Ablation	F-test Result
-1900	F(1, 22265292) = 7446.00, p < .001
-1910	F(1, 22265292) = 5654.92, p < .001
-1920	F(1, 22265292) = 1924.66, p < .001
-1930	F(1, 22265292) = 0.02, p = 0.88
-1940	F(1, 22265292) = 0.30, p = 0.58
-1950	F(1, 22265292) = 829.21, p < .001
-1960	F(1, 22265292) = 10396.38, p < .001
-1970	F(1, 22265292) = 6933.49, p < .001
-1980	F(1, 22265292) = 84397.30, p < .001
-1990	F(1, 22265292) = 200203.53, p < .001

Table 14

F-test results for ablation models using N-gram Fiction, with the 35–50 y.o. data. F-tests compare each ablated model against the full model.

Ablation	F-test Result
-1900	F(1, 21445111) = 6978.86, p < .001
-1910	F(1, 21445111) = 4920.91, p < .001
-1920	F(1, 21445111) = 2169.75, p < .001
-1930	F(1, 21445111) = 5.35, p = 0.02
-1940	F(1, 21445111) = 6.03, p = 0.01
-1950	F(1, 21445111) = 925.16, p < .001
-1960	F(1, 21445111) = 9580.19, p < .001
-1970	F(1, 21445111) = 6092.96, p < .001
-1980	F(1, 21445111) = 71582.65, p < .001
-1990	F(1, 21445111) = 157680.97, p < .001

Table 15

F-test results for ablation models using N-gram Fiction, with the 50–90 y.o. data. F-tests compare each ablated model against the full model.

Ablation	F-test Result
-1900	F(1, 19793799) = 8427.66, p < .001
-1910	F(1, 19793799) = 3462.03, p < .001
-1920	F(1, 19793799) = 3060.63, p < .001
-1930	F(1, 19793799) = 265.22, p < .001
-1940	F(1, 19793799) = 2023.01, p < .001
-1950	F(1, 19793799) = 1102.31, p < .001
-1960	F(1, 19793799) = 1122.88, p < .001
-1970	F(1, 19793799) = 33.48, p < .001
-1980	F(1, 19793799) = 816.55, p < .001
-1990	F(1, 19793799) = 46751.02, p < .001

Table 16

F-test results for ablation models using COHA Lemma, with the 20–35 y.o. data. F-tests compare each ablated model against the full model.

Ablation	F-test Result
-1900	F(1, 19517910) = 8805.29, p < .001
-1910	F(1, 19517910) = 3067.43, p < .001
-1920	F(1, 19517910) = 2465.13, p < .001
-1930	F(1, 19517910) = 324.73, p < .001
-1940	F(1, 19517910) = 2090.45, p < .001
-1950	F(1, 19517910) = 734.12, p < .001
-1960	F(1, 19517910) = 968.83, p < .001
-1970	F(1, 19517910) = 190.18, p < .001
-1980	F(1, 19517910) = 696.55, p < .001
-1990	F(1, 19517910) = 38819.23, p < .001

Table 17

F-test results for ablation models using COHA Lemma, with the 35–50 y.o. data. F-tests compare each ablated model against the full model.

Ablation	F-test Result
-1900	F(1, 18744680) = 7838.81, p < .001
-1910	F(1, 18744680) = 2661.88, p < .001
-1920	F(1, 18744680) = 2264.23, p < .001
-1930	F(1, 18744680) = 203.98, p < .001
-1940	F(1, 18744680) = 2090.95, p < .001
-1950	F(1, 18744680) = 661.56, p < .001
-1960	F(1, 18744680) = 448.19, p < .001
-1970	F(1, 18744680) = 114.70, p < .001
-1980	F(1, 18744680) = 608.36, p < .001
-1990	F(1, 18744680) = 30084.40, p < .001

Table 18

F-test results for ablation models using COHA Lemma, with the 50–90 y.o. data. F-tests compare each ablated model against the full model.

Ablation	Changed?	Neighbor	Older Adults (SE)	Younger Adults (SE)
1990	Changed	1990s	0.34 (1.53)	0.07 (1.15)
1980	Changed	1990s	-153.34 (17.38)	-140.14 (16.66)
1970	Changed	1990s	-1.92 (3.28)	-0.15 (1.65)
1960	Changed	1990s	-10.88 (5.11)	-5.42 (3.5)
1950	Changed	1990s	-20.52 (7.38)	-21.13 (7.08)
1940	Changed	1990s	-15 (5.81)	-19.16 (6.63)
1990	Changed	1950s	-29.25 (7.45)	-28.84 (7.98)
1980	Changed	1950s	-9.12 (4.37)	-4.03 (3.29)
1970	Changed	1950s	0.09 (0.81)	1.56 (1.22)
1960	Changed	1950s	-12.72 (4.98)	-2.26 (2.67)
1950	Changed	1950s	-41.63 (9.1)	-35.03 (8.39)
1940	Changed	1950s	-140.53 (18.02)	-120.14 (16.89)

Table 19

Expected log predictive density (ELPD) differences for the ablated models using N-gram All, relative to the full model. The standard error for the ELPD is in parenthesis after the ELPD difference. This table shows the subset for the Changed terms.

Ablation	Changed?	Neighbor	Older Adults (SE)	Younger Adults (SE)
1990	Unchanged	1950s	1.21 (0.98)	-0.36 (1.56)
1980	Unchanged	1950s	-11.38 (5.05)	-9.48 (4.43)
1970	Unchanged	1950s	-0.69 (2.03)	-0.8 (1.46)
1960	Unchanged	1950s	-1.01 (1.75)	0.65 (0.59)
1950	Unchanged	1950s	-0.41 (2.45)	-12.7 (5.31)
1940	Unchanged	1950s	-30.36 (9.51)	-23.46 (7.92)
1990	Unchanged	1990s	0.71 (0.52)	0.83 (0.42)
1980	Unchanged	1990s	-3.25 (2.56)	-12.97 (5.46)
1970	Unchanged	1990s	0.83 (0.36)	0.31 (1.92)
1960	Unchanged	1990s	-1.59 (1.92)	-0.12 (1.23)
1950	Unchanged	1990s	-17.5 (6.43)	-18.92 (6.6)
1940	Unchanged	1990s	-0.35 (2.32)	-0.49 (2.1)

Table 20

Expected log predictive density (ELPD) differences for the ablated models using N-gram All, relative to the full model. The standard error for the ELPD is in parenthesis after the ELPD difference. This table shows the subset for the Unchanged terms.

Ablation	Changed?	Neighbor	Older Adults (SE)	Younger Adults (SE)
1990	Changed	1990s	-114.17 (16.83)	-108.23 (16.06)
1980	Changed	1990s	0.2 (1.08)	0.01 (1.17)
1970	Changed	1990s	0.91 (0.57)	0.39 (0.89)
1960	Changed	1990s	-1.43 (2.12)	-0.77 (2.13)
1950	Changed	1990s	-6.05 (3.18)	-6.97 (3.37)
1940	Changed	1990s	-0.13 (1.63)	-0.12 (0.83)
1990	Changed	1950s	-59.52 (11.23)	-49.03 (10.04)
1980	Changed	1950s	-31.8 (8.49)	-25.72 (8.26)
1970	Changed	1950s	1.42 (1.01)	1.38 (0.78)
1960	Changed	1950s	0.49 (1)	0.37 (1.71)
1950	Changed	1950s	1.73 (0.62)	0.95 (0.71)
1940	Changed	1950s	-8.1 (4.92)	-3.9 (3.64)

Table 21

Expected log predictive density (ELPD) differences for the ablated models using N-gram Fiction, relative to the full model. The standard error for the ELPD is in parenthesis after the ELPD difference. This table shows the subset for the Changed terms.

Ablation	Changed?	Neighbor	Older Adults (SE)	Younger Adults (SE)
1990	Unchanged	1950s	-8.25 (4.46)	-3.72 (3.72)
1980	Unchanged	1950s	-17.7 (5.8)	-20.87 (6.69)
1970	Unchanged	1950s	-0.58 (1.69)	0.91 (1.12)
1960	Unchanged	1950s	-0.44 (0.83)	0.16 (0.73)
1950	Unchanged	1950s	0.26 (0.4)	0.37 (0.47)
1940	Unchanged	1950s	0.07 (0.65)	0.23 (0.54)
1990	Unchanged	1990s	-20.98 (6.49)	-25.62 (7.1)
1980	Unchanged	1990s	-3.44 (2.58)	-2.55 (2.79)
1970	Unchanged	1990s	-2.82 (2.44)	-3.13 (2.84)
1960	Unchanged	1990s	-0.58 (2.05)	-2.71 (3.07)
1950	Unchanged	1990s	-3.15 (2.73)	0.3 (1.15)
1940	Unchanged	1990s	-5.18 (3.87)	-2.63 (2.42)

Table 22

Expected log predictive density (ELPD) differences for the ablated models using N-gram Fiction, relative to the full model. The standard error for the ELPD is in parenthesis after the ELPD difference. This table shows the subset for the Unchanged terms.

Ablation	Changed?	Neighbor	Older Adults (SE)	Younger Adults (SE)
1990	Changed	1990s	-132.18 (16.69)	-138.61 (17.11)
1980	Changed	1990s	0.29 (1.57)	-2.2 (2.74)
1970	Changed	1990s	-3.96 (3.27)	-4.04 (3.5)
1960	Changed	1990s	0.92 (1.03)	1.55 (0.79)
1950	Changed	1990s	0.26 (2.44)	-2.07 (3.53)
1940	Changed	1990s	1.81 (0.66)	0.75 (0.48)
1990	Changed	1950s	-3.55 (2.59)	-0.7 (2.42)
1980	Changed	1950s	-7.52 (4.58)	-7.79 (4.56)
1970	Changed	1950s	0.39 (0.8)	2.04 (0.73)
1960	Changed	1950s	-4.09 (3.03)	1.32 (1.84)
1950	Changed	1950s	-4.57 (3.2)	-2.45 (3.16)
1940	Changed	1950s	0.48 (0.37)	1.2 (1.3)

Table 23

Expected log predictive density (ELPD) differences for the ablated models using COHA Lemma, relative to the full model. The standard error for the ELPD is in parenthesis after the ELPD difference. This table shows the subset for the Changed terms.

Ablation	Changed?	Neighbor	Older Adults (SE)	Younger Adults (SE)
1990	Unchanged	1950s	-13.31 (4.92)	-23.41 (6.5)
1980	Unchanged	1950s	1.08 (1.38)	0.58 (0.7)
1970	Unchanged	1950s	-11.15 (4.82)	-6.24 (3.47)
1960	Unchanged	1950s	1 (1.26)	1.56 (0.63)
1950	Unchanged	1950s	-1.6 (1.9)	0.89 (0.37)
1940	Unchanged	1950s	1.05 (1.53)	-6.37 (3.84)
1990	Unchanged	1990s	-15.15 (5.7)	-19.09 (6.83)
1980	Unchanged	1990s	0.93 (0.42)	0.8 (0.61)
1970	Unchanged	1990s	1.04 (0.62)	-0.35 (2.33)
1960	Unchanged	1990s	0.58 (0.56)	0.85 (0.31)
1950	Unchanged	1990s	0.2 (0.56)	-0.62 (1.5)
1940	Unchanged	1990s	-0.16 (1.61)	0.42 (0.94)

Table 24

Expected log predictive density (ELPD) differences for the ablated models using COHA Lemma, relative to the full model. The standard error for the ELPD is in parenthesis after the ELPD difference. This table shows the subset for the Unchanged terms.
